# Characterisation of Mycelium-Based Biomaterials Derived from Diverse Mushroom Species for Sustainable Applications

**DOI:** 10.3390/jof12080617

**Published:** 2026-08-16

**Authors:** Sabin Khyaju, Kevin D. Hyde, Kitiphong Khongphinitbunjong, Sitthi Duangphet, Thatsanee Luangharn

**Affiliations:** 1 Center of Excellence in Fungal Research, Mae Fah Luang University, Chiang Rai 57100, Thailand; khyajusabin@gmail.com (S.K.);; 2 School of Science, Mae Fah Luang University, Chiang Rai 57100, Thailand; 3 Mushroom Research Foundation, Bandu, Chiang Rai 57100, Thailand; 4 Center of Innovative Materials for Sustainability, Mae Fah Luang University, Chiang Rai 57100, Thailand

**Keywords:** biocircular economy, biodegradable, fungal applications, mycelium-based biomaterials

## Abstract

Mushroom mycelium-based biomaterials are eco-friendly, biodegradable, cheap, and sustainable materials. In this study, ten wild mushroom species were collected from tropical forests in Chiang Mai and Chiang Rai provinces, and two commercial mushroom species were obtained from a local market in Chiang Rai Province, Thailand. Mushroom specimens were identified based on morphological characteristics and phylogenetic analysis of the internal transcribed spacer (ITS) region. All fungal species were determined to use suitable agar media (potato dextrose agar; malt extract agar), liquid media (malt extract broth), and agricultural substrate (rubber sawdust size ≤ 2 mm) for mushroom mycelial growth. Mechanical property evaluation using Ashby’s chart indicated that the resulting mushroom mycelium-based biomaterials have the potential to substitute for certain types of synthetic foam. A biodegradability test was conducted through soil burial of samples for 90 days, which demonstrated cumulative weight loss exceeding 60%, thereby confirming biodegradability. Furthermore, a new approach for maintaining mycelial viability was developed and validated, addressing the challenge of maintaining vigorous mycelium. Notably, this study presents the first comprehensive report on the application of *Coriolopsis brunneoleuca*, *Ganoderma tropicum*, *Hexagonia* sp., *Microporus xanthopus*, *Trametes polyzona*, and *T. sanguinea* in mushroom mycelium-based biomaterial production, including material development, characterisation, and prototype fabrication. Based on the material properties and successfully developed prototypes, the mushroom mycelium-based biomaterials produced in this study are potentially suitable for applications in packaging, indoor products, construction, and insulation purposes, as a viable alternative to conventional synthetic materials.

## 1. Introduction

Mushroom mycelium-based biomaterials are innovative and sustainable alternative materials to conventional synthetic plastics [1]. Mushroom mycelium-based biomaterials offer multiple benefits, including energy-efficient production, cost-effectiveness, complete biodegradability, potential for carbon dioxide sequestration, sustainable sourcing, lightweight, low density, non-toxic, and remarkable suitability for the packaging and construction industries [2,3]. Mushroom mycelium-based biomaterials have been produced either as pure mycelium materials or in combination with lignocellulosic substrates, depending on the purpose of the final products. In recent years, the utilisation of mycelium-derived biomaterials has elevated from experimental level to industrial and commercial companies, such as Ecovative Design, Mogu, and Mycoworks [4,5].

Despite the immense advantages of mushroom mycelium-based biomaterials, only a limited number of fungal species, mostly white-rot fungi, have been studied for biomaterial investigation. Screening fungal species is an important criterion, as mycelial density, mycelial structure, and growth rate directly affect biomaterial properties [6]. Based on a survey of available research on biomaterials conducted up to 2024, fungal species in Polyporales rank first for desirable inherent properties [7]. Intensive exploration of a suitable combination of a fungal candidate, substrate, and development method is always a primary concern for addressing the limitations of biomaterials. Recent studies indicate that potential fungal species for mycelium-based biomaterials may not yet have been identified and investigated [7].

The objective of this work is to investigate twelve fungal strains, ten wild and two commercial, in biomaterial development and characterisation. This study analyses physical, mechanical, chemical, and hydrodynamic properties of developed biomaterials and comprehensively reports them. Additionally, developing prototypic biomaterials could demonstrate the potential for a wide range of applications that benefit human daily life.

## 2. Materials and Methods

### 2.1. Specimen Collection and Isolation

**Figure 1:**
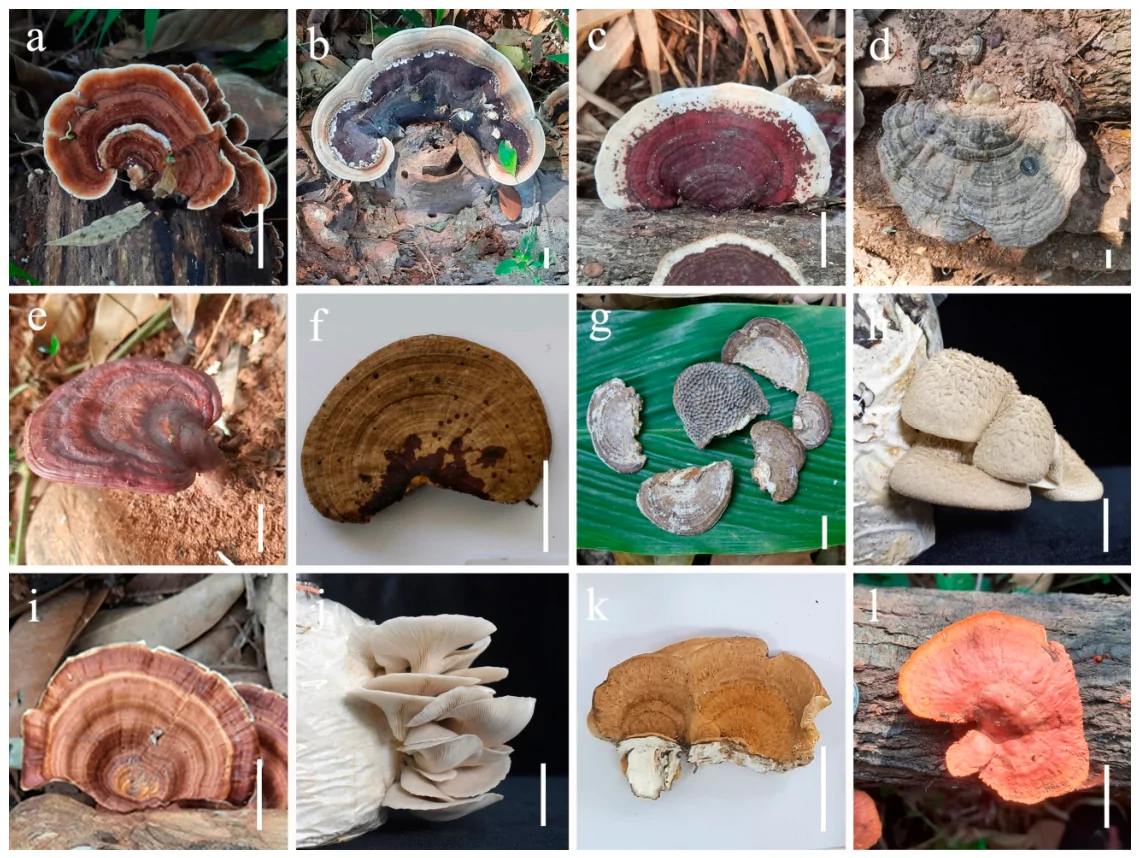
Basidiomata of fungal strains used in this study. (**a**) *Coriolopsis brunneoleuca* (Co); (**b**) *Earliella scabrosa* (Ea1); (**c**) *E. scabrosa* (Ea2); (**d**) *Ganoderma australe* (Ga1); (**e**) *G. tropicum* (Ga2); (**f**) *Hexagonia* sp. (He1); (**g**) *H.* sp. (He2); (**h**) *Lentinus squarrosulus* (Le); (**i**) *Microporus xanthopus* (Mi); (**j**) *Pleurotus ostreatus* (Pl); (**k**) *Trametes polyzona* (Tr1); (**l**) *T. sanguinea* (Tr2). Scale bar: a–l = 2 cm.

Fresh specimens of ten wild fungal strains growing on decaying logs were collected from mixed deciduous forests in Chiang Rai and Chiang Mai provinces of Thailand. In addition, two commercially cultivated species were obtained from a local market in Chiang Rai Province (Figure 1, Table 1). In the field, the specimens were air-dried at 55 °C for 48 h to ensure complete dehydration [8]. Dried specimens were preserved in paper bags and later used for molecular and phylogenetic studies in the laboratory. For culture isolation, fresh tissue from the samples was aseptically transferred onto potato dextrose agar and incubated at 25 °C in the dark. After 3–5 days, cultures were subcultured onto a fresh PDA plate to obtain a pure culture, which was then maintained at 4 °C for further studies.

### 2.2. Species Identification

Genomic DNA was extracted from pure cultures using the High Pure PCR Template Preparation Kit (Roche, Basel, Switzerland). DNA quality and concentration were assessed using a NanoDrop One Microvolume UV–Vis spectrophotometer (Thermo Scientific, Waltham, MA, USA). The internal transcribed spacer (ITS) region was amplified using the primers ITS1/ITS4 [9]. Polymerase chain reaction (PCR) was performed in a total volume of 25 µL, containing 12.5 µL of 2× Power Taq PCR Master Mix, 1 µL of each primer (20 µM), 1 µL of genomic DNA, and 9.5 µL of deionised water. Amplifications were performed using an Eppendorf Mastercycler (Eppendorf AG 22331, Hamburg, Germany) under the following thermal cycling conditions: initial denaturation at 94 °C for 3 min, followed by 35 cycles of 95 °C for 30 s, 55 °C for 1 min, and 72 °C for 1 min; and final extension at 72 °C for 10 min. PCR products were sequenced by SolGent Co., Ltd., Yuseong-gu, Daejeon, Republic of Korea. The final consensus sequences were subjected to online BLAST (https://blast.ncbi.nlm.nih.gov/Blast.cgi, accessed on 12 August 2026) searches against the National Center for Biotechnology Information (NCBI) database for species identification. Pure cultures were deposited at Mae Fah Luang University Culture Collection (MFLUCC).

### 2.3. Evaluation of Culture Media for Mycelial Growth

Mycelial growth was evaluated on MEA and PDA. All equipment and media were sterilised at 121 °C and 15 psi for 20 min. Mycelial plugs (5 mm in diam.) obtained from 10-day-old PDA cultures were transferred onto the test media and incubated at 25 °C in darkness for 12 days. Radial growth was recorded daily following the method described by [10]. The mycelium density was evaluated as very scanty (1+), scanty (2+), moderate (3+), somewhat abundant (4+), or abundant (5+) [10]. All treatments were performed in five replicates. Confidence interval was calculated as: CI = $\bar{x}$ ± z.s/√n, where $\bar{x}$ = sample mean, z = confidence level value, s = sample standard deviation, and n = sample size.

### 2.4. Evaluation of Mycelial Growth on Lignocellulose Substrates

Substrate bags were prepared following [11] with modifications. The substrate consists of sawdust (≤2 mm particle size, 80% *w*/*w*), supplemented with rice bran (10%), yeast extract (4%), molasses (4%), calcium carbonate (1.5%), and gypsum (0.5%). Moisture content and pH were adjusted to 50–60% and 5.5–6.5, respectively. Approximately 30 g of substrate was placed in a glass petri dish (9 cm diam.), packed into polypropylene bags, and sterilised at 121 °C for 60 min. After cooling, each plate was inoculated with 5 mm mycelial plugs from 10-day-old cultures and incubated at 25 °C in darkness. Each treatment was performed in five replicates. Mycelial growth (colony diam.) was measured every two days until full colonisation, and mycelial density was recorded on day 14.

### 2.5. Preparation of Pure Mycelium Mat Samples

Pure mycelium was cultivated in two broth media: malt extract broth and potato dextrose broth. Mycelial plugs (1 cm in diam.) from actively growing cultures were inoculated into 150 mL of each medium and incubated at 25 °C in the dark for 30, 45, and 60 days, with three replicates per treatment, following a modification of [12]. After each incubation period, mycelial mats were harvested, rinsed with reverse osmosis (RO) water to remove residual broth, and dried at 40 °C for 48 h or until a constant weight was achieved.

### 2.6. Preparation of Mycelium-Based Biomaterials Test Samples

Test samples were prepared from selected fungal species through a series of steps, including spawn preparation, substrate bag formulation, biomaterial fabrication, and denaturation, as described below.

#### 2.6.1. Preparation of Spawn

Spawn was prepared following [8] with modifications. Sorghum grains (*Sorghum bicolor*) were washed and soaked overnight in tap water and subsequently boiled for 15 min. After cooling and air-drying at room temperature to remove excess moisture, approximately 150 g of prepared grains was transferred into sterile glass bottles and autoclaved at 121 °C for 30 min. Once cooled, mycelial plugs from each fungal strain were aseptically inoculated into the bottles and incubated in the dark at 28 °C for 25 days or until full colonisation was achieved.

#### 2.6.2. Preparation of Mycelium Substrate Bags

Substrate bags were prepared following the method of [11] with modifications. The substrate consisted of rubber sawdust (80% *w*/*w*), supplemented with rice bran (10%), yeast extract (4%), molasses (4%), calcium carbonate (1.5%), and gypsum (0.5%). All ingredients were manually mixed to achieve a moisture content of 55–60%. A total of 700 g of the mixture was then packed into polypropylene bags, sealed with plastic rings and lids, and sterilised at 121 °C for 60 min. After cooling, each bag was aseptically inoculated with 20 g of spawn and incubated in darkness at 25 ± 1 °C until complete colonisation was achieved.

#### 2.6.3. Preparation of Moulds

Moulds were fabricated according to the test specifications. For compression strength testing, acrylic cubic moulds (50 × 50 × 50 mm) were prepared following [13]. Flexural strength moulds were prepared as cuboids (120 × 50 × 50 mm). Impact test moulds were prepared as cuboids (63.5 × 12.7 × 12.7 mm), conforming to American Society for Testing and Materials (ASTM) standards [14]. For the flammability test, cuboids (125 × 13 × 5 mm) were prepared. Circular samples (90 mm diam) for FTIR spectroscopy were prepared using glass petri dishes.

#### 2.6.4. Fabrication of Mycelium-Based Biomaterials

Following complete colonisation, the mycelium–substrate mass was manually broken apart, transferred into sterile acrylic moulds, and pressed to form test samples. The moulded materials were incubated for 7–10 days, and then, the samples were removed from the moulds and incubated for an additional 7–10 days to allow mycelial growth on surfaces previously in contact with the mould. All the equipment was washed with tap water and sterilised by heat treatment in a hot-air oven at 60 °C overnight. All procedures were conducted under aseptic conditions in a laminar flow hood to prevent contamination.

#### 2.6.5. Denaturation

**Figure 2:**
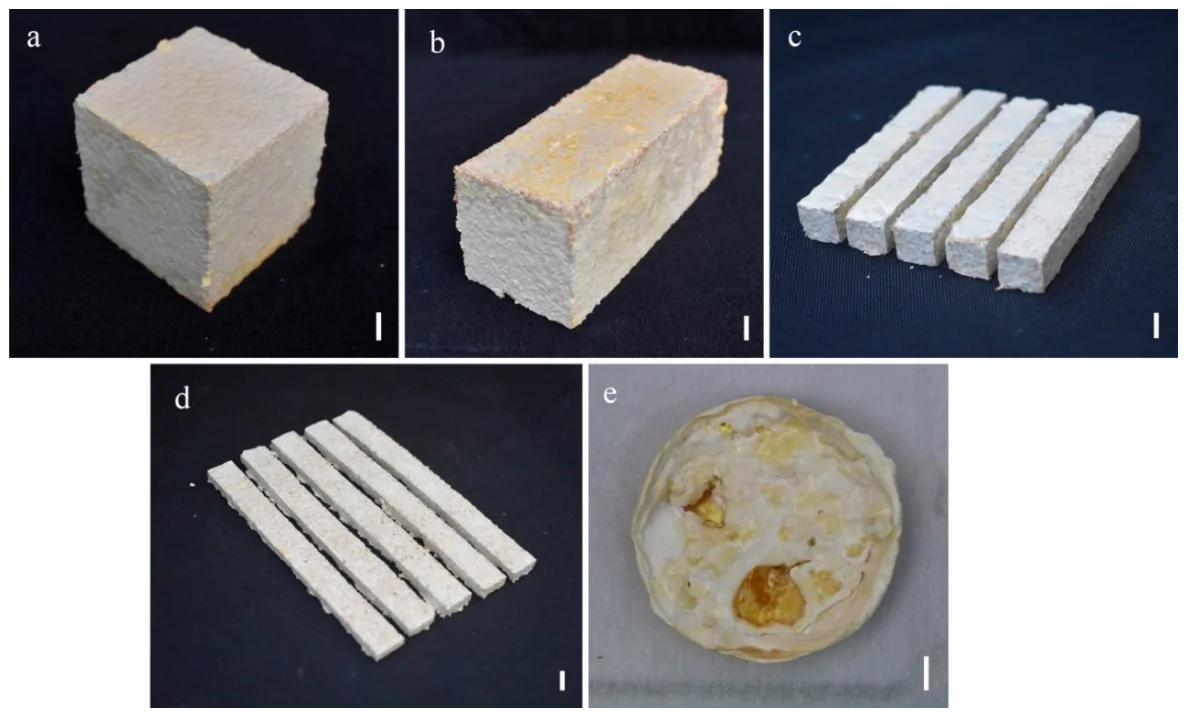
Representative mycelium-based samples used in this study were produced from *Trametes polyzona* (Tr1). (**a**) Sample prepared for moisture content, dry density, volumetric shrinkage, compression strength, and water absorption tests; (**b**) sample for flexural strength, SEM analysis, chemical tests (pH, organic matter, nitrogen, electrical conductivity), and soil burial degradability tests; (**c**) sample for impact strength test; (**d**) sample for flammability test; (**e**) sample for TGA and FTIR analyses. Scale bar: a–e = 1 cm.

Fully colonised test samples were deactivated by drying in a hot-air oven at 60 °C for 48 h [15]. After cooling to room temperature, the samples (Figure 2) were subjected to physical, mechanical, hydrodynamic, and chemical analyses.

### 2.7. Evaluation of Physical Properties of Mycelium-Based Biomaterial

#### 2.7.1. Biomass of Mycelium Mats

Mycelial biomass was determined by harvesting pure mycelium mats cultivated on the three best-performing media. The mats were rinsed with RO water for 1–2 min and oven-dried at 45 °C for 24 h until a constant weight was achieved [16]. Dry weight was recorded on days 30, 45, and 60.

#### 2.7.2. Moisture Content and Dry Density

The dry density of mycelium-based biomaterials was determined according to ISO 9427:2003 [8]. Density was calculated as the ratio of oven-dry mass to volume using cubic test samples (5 × 5 × 5 cm). All measurements were conducted in five replicates.

#### 2.7.3. Volumetric Shrinkage

Volumetric shrinkage was determined using fully colonised mushroom mycelium-based biomaterial cubes (5 × 5 × 5 cm) following [17]. The percentage of shrinkage was calculated as: shrinkage (%) = (V_1_ − V_2_)/ V_1_ × 100, where V_1_ = moist volume of the sample and V_2_ = dry volume. The test was conducted in five replicates.

### 2.8. Evaluation of Mechanical Properties of Mycelium-Based Biomaterial

#### 2.8.1. Compression Strength

**Figure 3:**
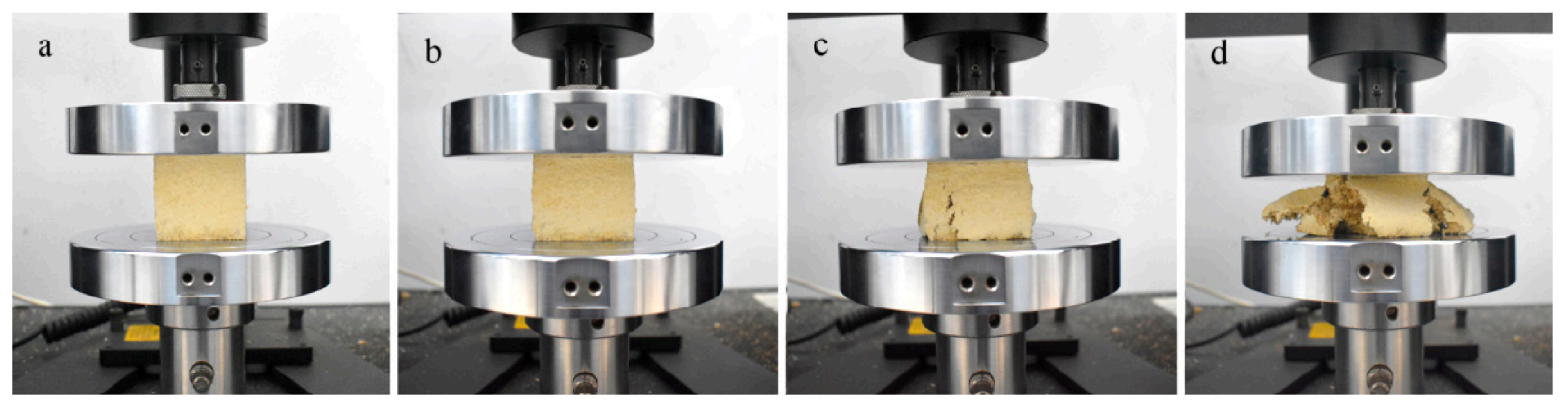
Compression strength test of mycelium-based biomaterial sample of *Trametes polyzona* (Tr1). (**a**) Before testing; (**b**) bulging; (**c**) initial cracking; (**d**) complete crushing.

Compression strength was determined according to ASTM D3501-94 following [17], using a Universal Testing Machine (UTM; Instron Model 5566, Instron Corporation, Norwood, MA, USA) (Figure 3). Tests were conducted using a 10 kN capacity load bench and a 10 kN load cell under ambient conditions (25 °C, ~50% relative humidity) at a displacement rate of 5 mm min^-1^. Five replicates were tested per sample. Compression strength (kPa) was calculated from the load-displacement data as: compressive stress (σ) = F/A and strain (ε) = (∆L/Lo), where F = compressive force (N), A = original cross-sectional area (m^2^), ΔL = displacement of the loading surfaces (m), and Lo = original height of the specimen (m).

#### 2.8.2. Flexural Strength

**Figure 4:**
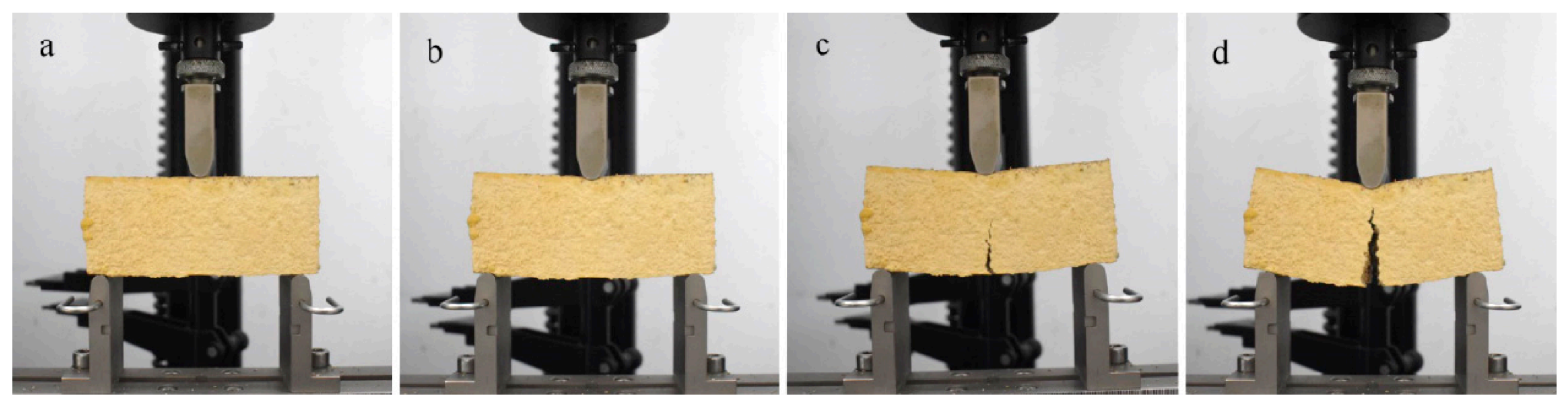
Flexural strength test of mycelium-based biomaterial sample of *Trametes polyzona* (Tr1). (**a**) Before testing; (**b**) bending; (**c**) initial cracking; (**d**) complete breakage.

Flexural strength was determined according to ASTM D790-10 following [18], using a Universal Testing Machine ( UTM; Instron Model 5566, Instron Corporation, Norwood, MA, USA) (Figure 4). Tests were conducted at a cross-head speed of 2 mm min^−1^ with a clamp support span of 100 mm. Five replicates were tested per sample. Flexural strength (kPa) was calculated from the load-displacement data as: flexural stress (σf) = 3FL/(2bd^2^), where F = applied load at fracture (N), L = support span (m), b = specimen width (m), and d = specimen thickness (m).

#### 2.8.3. Impact Strength

Impact strength was determined using the Charpy impact test according to ASTM D256 following [14], using an impact pendulum machine (Instron; Model CEAST 9050, Turin, Italy). Samples were subjected to a pendulum strike until fracture. Each strain was tested with five replicates. Impact strength (IS) was calculated as: IS = K/A, where K = energy required to fracture the sample (kJ) and A = cross-sectional area (m^2^).

### 2.9. Evaluation of Hydrodynamic Properties of Mycelium-Based Biomaterials

#### 2.9.1. Water Absorption

Water absorption was determined according to ASTM C272/C272M-18 following Attias et al. [19] with modifications. Mushroom mycelium-based biomaterial samples (50 × 50 × 50 mm) were partially submerged in water at room temperature (25 °C, ~50% RH). Weight of the samples was measured every 24 h interval. The water absorption rate was calculated based on the change in weight relative to the initial dry weight. Five replicates were performed per treatment.

#### 2.9.2. Water Contact Angle

Water contact angle was measured using a contact angle meter (Kino SL200KS, Puditec, Bangkok, Thailand) following Antinori et al. [20]. A 5 μL droplet of deionised water was statically deposited on the sample surfaces, and side-view images were captured at 10 and 60 s. Fifteen replicates were performed per treatment.

### 2.10. Evaluation of Chemical Properties of Mycelium-Based Biomaterial

Mushroom mycelium-based biomaterial samples were ground into powder form using a blender and sieved to obtain particles (˂2 mm). The pH was determined by mixing 5 g of the sample with 50 mL of Milli-Q water for 1 h and measuring with a calibrated pH meter [6]. Organic matter (%) and nitrogen (%) contents were determined following the Walkley–Black method [21] and the Kjeldahl method [22], respectively.

### 2.11. Scanning Electron Microscopy

Mushroom mycelium-based biomaterial samples (5 × 5 mm) were cut using a dissecting blade, mounted on stub adapters with double-sided carbon tape, and gold-coated for 2 min under high vacuum following [14]. Surface morphology was examined using a scanning electron microscope (SEM; TESCAN MIRA, 4th Generation, Tescan Orsay Holding, Brno-Kohoutovice, Czech Republic) operated at 5 kV with TESCAN’s Essence™ software (v 1.0.5.1). Both surfaces and cross-sectional characteristics were observed.

### 2.12. Thermogravimetric Analysis

Thermogravimetric analysis (TGA) was performed on approximately 8 mg of pure mycelium from each fungal strain using a thermal analyser (Mettler Toledo, TGA/DSC3+ HT, Mettler Toledo, Greifensee, Switzerland) following [23]. Samples were heated from 25 °C to 900 °C at a scanning rate of 20 °C min^−1^ under a constant nitrogen purge flow of 25 mL min^−1^.

### 2.13. Fourier Transform Infrared Spectroscopy

Fourier transform infrared (FTIR) spectroscopy was performed to characterise the chemical composition of pure mycelium samples using attenuated total reflectance (ATR-FTIR) spectroscopy (Nicolet iS50, Thermo Fisher Scientific Inc., Madison, WI, USA) [12]. FTIR spectra were recorded in the range of 4000−400 cm^−1^ at a resolution of 4 cm^−1^ with 32 scans.

### 2.14. Soil Burial Degradability

Biodegradability was assessed under soil burial conditions according to ISO 846:2000 [8] using fine-grained active soil collected from forest areas in Chiang Rai Province, Thailand. Soil was sieved to obtain particles ˂ 2 mm [14]. Test samples were buried for 90 days with three replicates. Test samples were retrieved at 15, 30, 45, 60, 75, and 90 days; cleaned; and oven-dried at 60 °C to constant weight. Weight loss was calculated as: WL (%) = (Wi − Wt)/Wi × 100, where WL = the percentage of weight loss (%), Wi = initial weight (g), and Wt = weight after the specified time (g).

### 2.15. Viability Maintenance of Fungal Species

The viability of the fungal strains was maintained through basidiomata formation [24]. Fresh basidiomata were produced using the same substrate composition employed for test sample preparation. Cultivation bags were initially incubated in darkness at 25 °C and then transferred to a fruiting chamber maintained at 25 ± 1 °C with 75–80% relative humidity and illumination. Resulting basidiomata were used for isolation and preparation of pure cultures for further study. Five cultivation bags were used per species.

### 2.16. Developing Prototypic Product

A biomaterial prototype insulation wall was fabricated using a mixture of mushroom mycelium and sawdust substrate. Blocks were prepared following the same procedure used for test sample development. Fully colonised blocks were dehydrated at 60 °C for 5 days until constant weight was achieved. Individual blocks (30 × 25 × 10 cm) were assembled into a large panel framework (120 × 75 × 10 cm) constructed from transparent acrylic sheets.

### 2.17. Data Collection and Statistical Analysis

Statistical analyses were performed using SPSS v.16.0. Data on mycelial growth rate, as well as physical, mechanical, hydrodynamic, chemical, and soil burial biodegradability properties, were analysed using one-way analysis of variance (ANOVA). An independent samples t-test was used to compare the means between two independent groups. When significant differences were detected (*p* < 0.05), multiple comparisons were conducted using Duncan’s multiple range test (DMRT).

## 3. Results

### 3.1. Effect of Culture Media on Mycelial Growth

**Figure 5:**
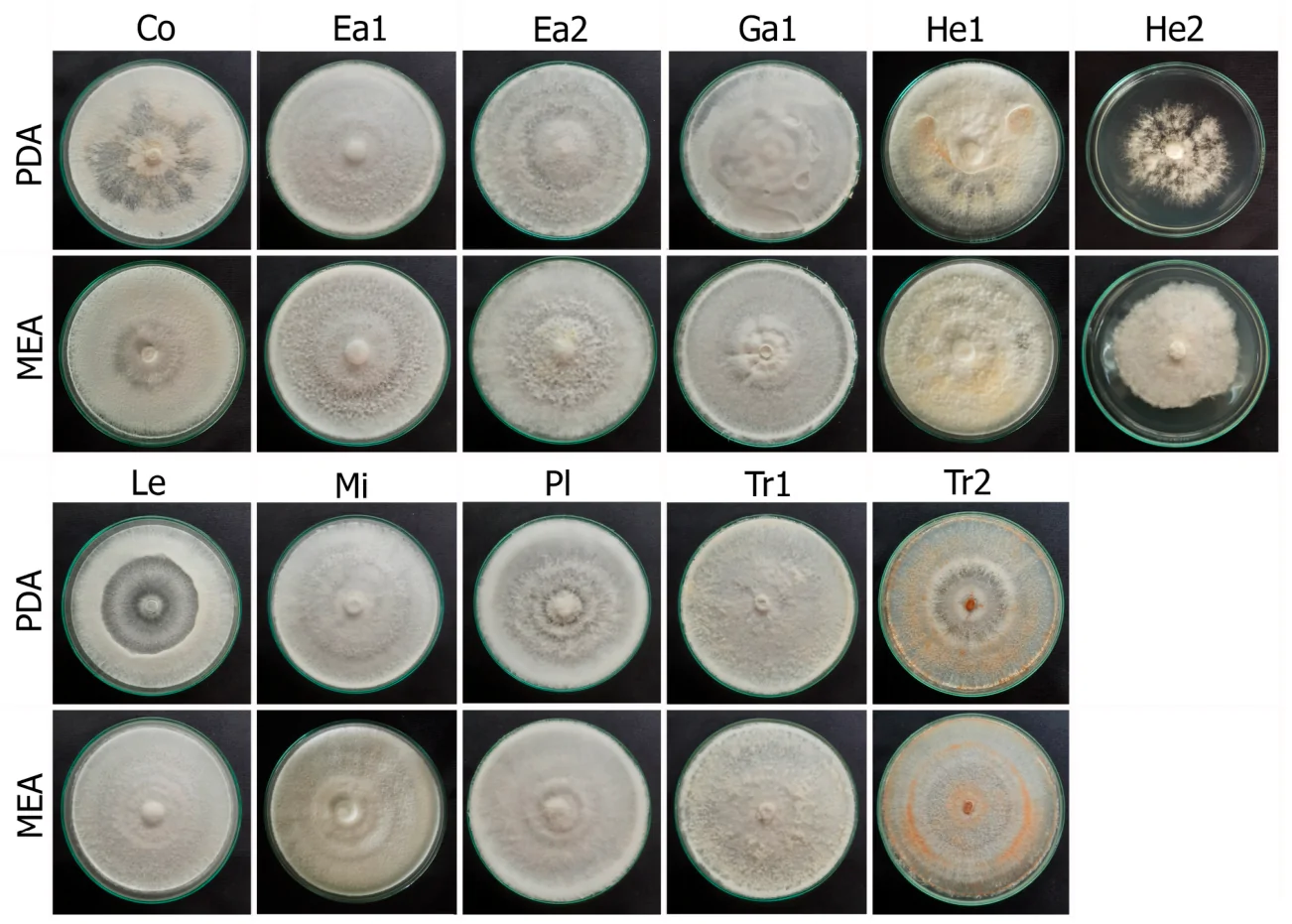
Morphology of mycelia of the fungal strains grown on PDA and MEA at day 14 after inoculation.

The mycelium growth rate and density of fungal strains cultivated in PDA and MEA are presented in Figure 5 and Table 2. Considerable variation in growth performance was observed among strains and between the two media. On PDA, the fastest growth rates were observed for *Trametes sanguinea* (13.58 mm/day) and *Lentinus squarrulosus* (13.07 mm/day), followed by *Microporus xanthopus* (12.77 mm/day) and *T. polyzona* (12.60 mm/day). On MEA, *T. polyzona* (14.22 mm/day) exhibited the fastest growth, followed by *L. squarrulosus* (14.08 mm/day). However, *Hexagonia* sp. (He2) exhibited the lowest growth rate, with 4.24 mm/day and 5.70 mm/day on PDA and MEA, respectively. In terms of colony density, both *Earliella scabrosa* strains, *Hexagonia* sp. (He1), and *Pleurotus ostreatus* exhibited the thickest mycelial density (5+) in both media. In Figure 5, mycelium is uniformly grown in *E. scabrosa*, *G. australe*, *Hexagonia* sp. (He1), *M. xanthopus*, *P. ostreatus*, and *T. polyzona*.

### 3.2. Mycelium Characteristics on Lignocellulose Substrates

**Figure 6:**
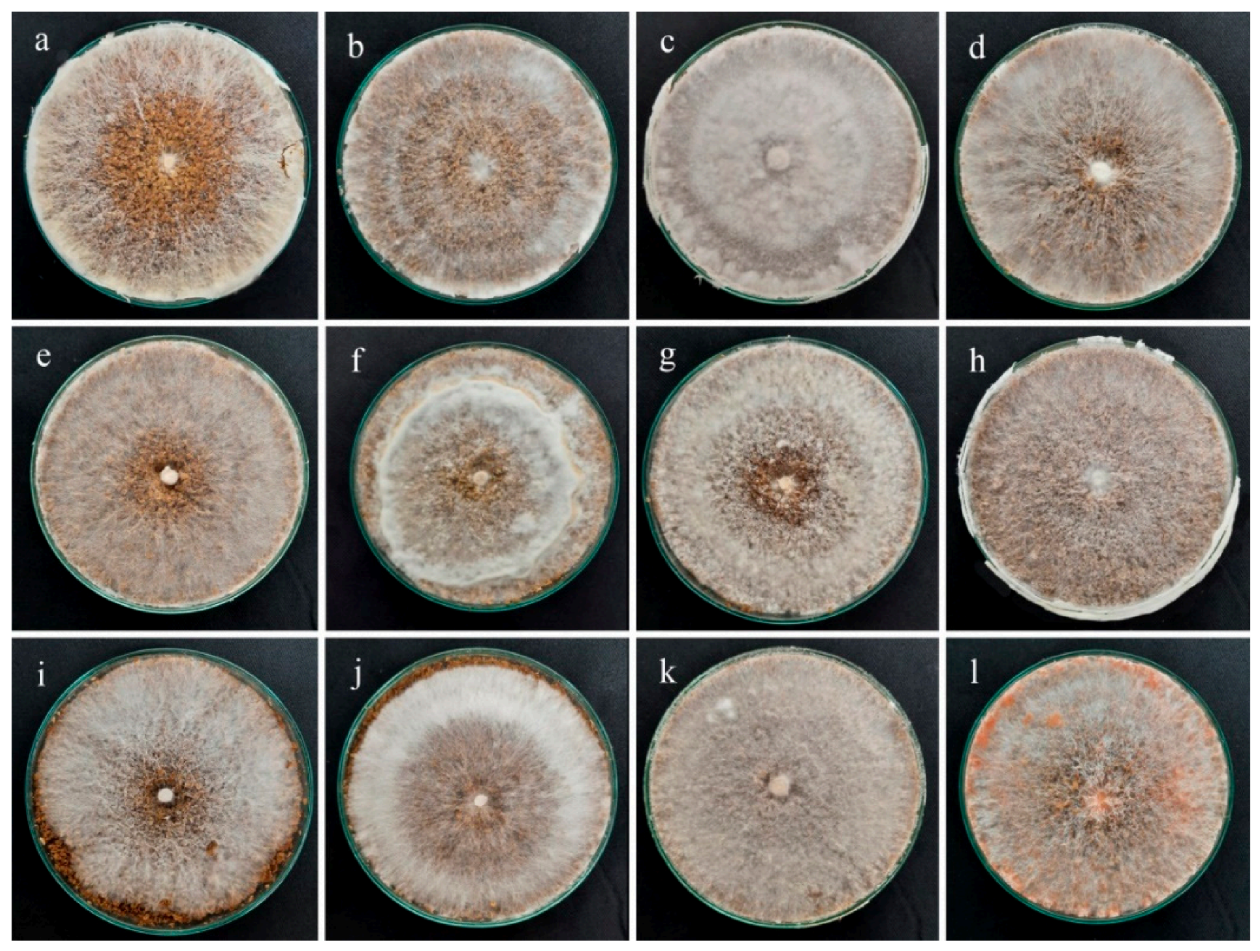
Mycelium growth of the studied fungal species on sawdust at day 30. (**a**) *Coriolopsis brunneoleuca*; (**b**) *Earliella scabrosa* (Ea1); (**c**) *E. scabrosa* (Ea2); (**d**) *Ganoderma austral*; (**e**) *G. tropicum*; (**f**) *Hexagonia* sp. (He1); (**g**) *Hexagonia* sp. (He2); (**h**) *Lentinus squarrosulus*; (**i**) *Microporus xanthopus*; (**j**) *Pleurotus ostreatus*; (**k**) *Trametes polyzona*; (**l**) *T. sanguinea*.

**Figure 7:**
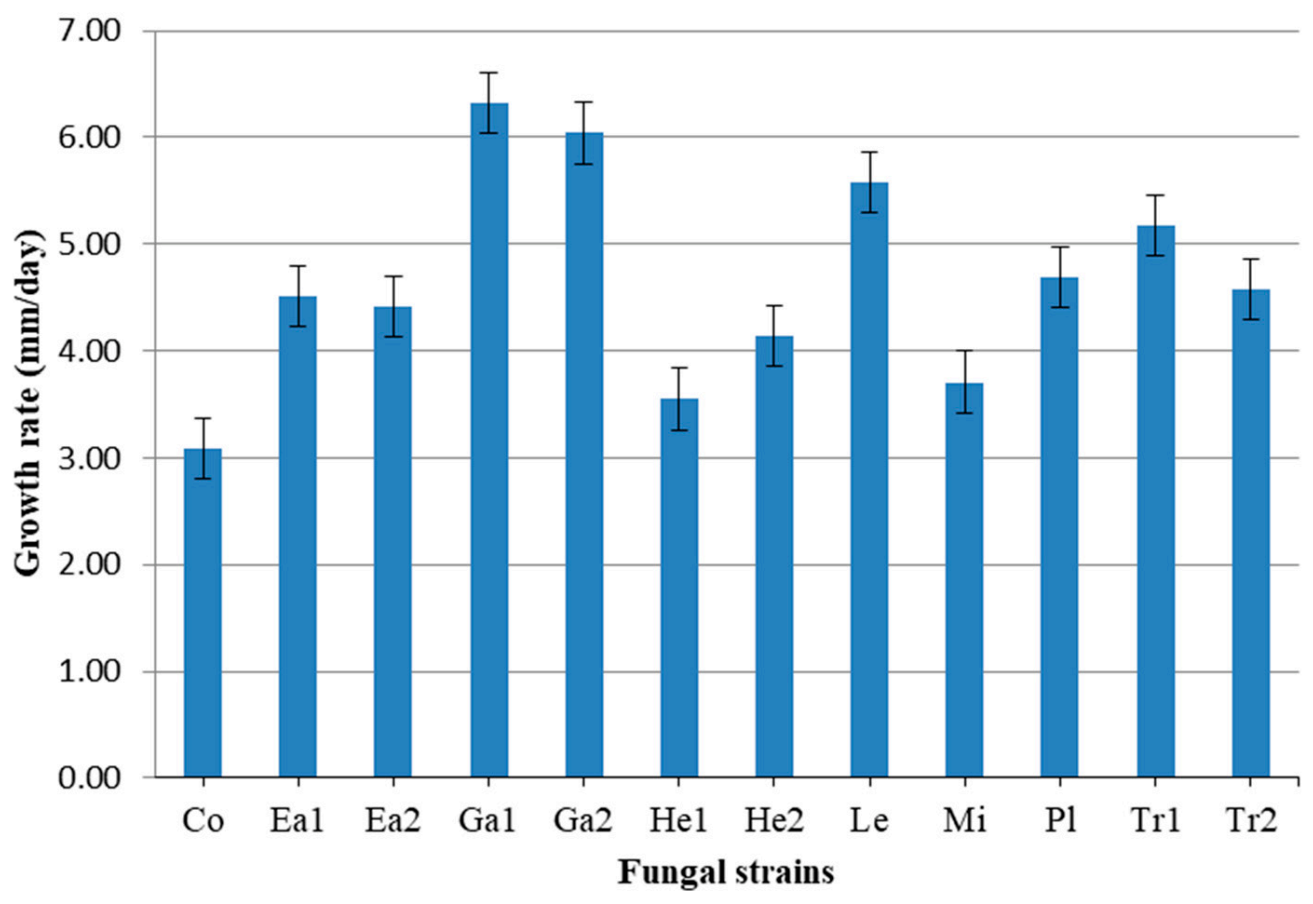
Mycelium growth rate of the selected fungal strains on sawdust. Error bars indicate the standard deviation of the means.

The visual representation of the mycelium growth of the studied fungal strains on rubber sawdust at day 30 is shown in Figure 6. The growth rate and surface morphology of mycelia in sawdust are provided in Figure 7 and Table 3. *Ganoderma australe* and *G. tropicum* exhibited the fastest growth rates, at 6.32 and 6.04 mm/day, respectively. *Coriolopsis brunneoleuca* showed the slowest mycelial growth rate, with 3.09 mm/day. *Earliella scabrosa* (Ea2), *Hexagonia* spp. (He1, He2), and *Pleurotus ostreatus* showed the thickest mycelial density. Both *Earliella* spp. and *Hexagonia* sp. (He1) showed a concentric mycelium pattern, while other strains showed white and uniform mycelium. Notably, orange pigmentation was observed on the mycelial surface of *Trametes sanguinea*.

### 3.3. Morphological Characteristics of Fungal Mycelia in Liquid Media

#### 3.3.1. Morphological Features in Potato Dextrose Broth

**Figure 8:**
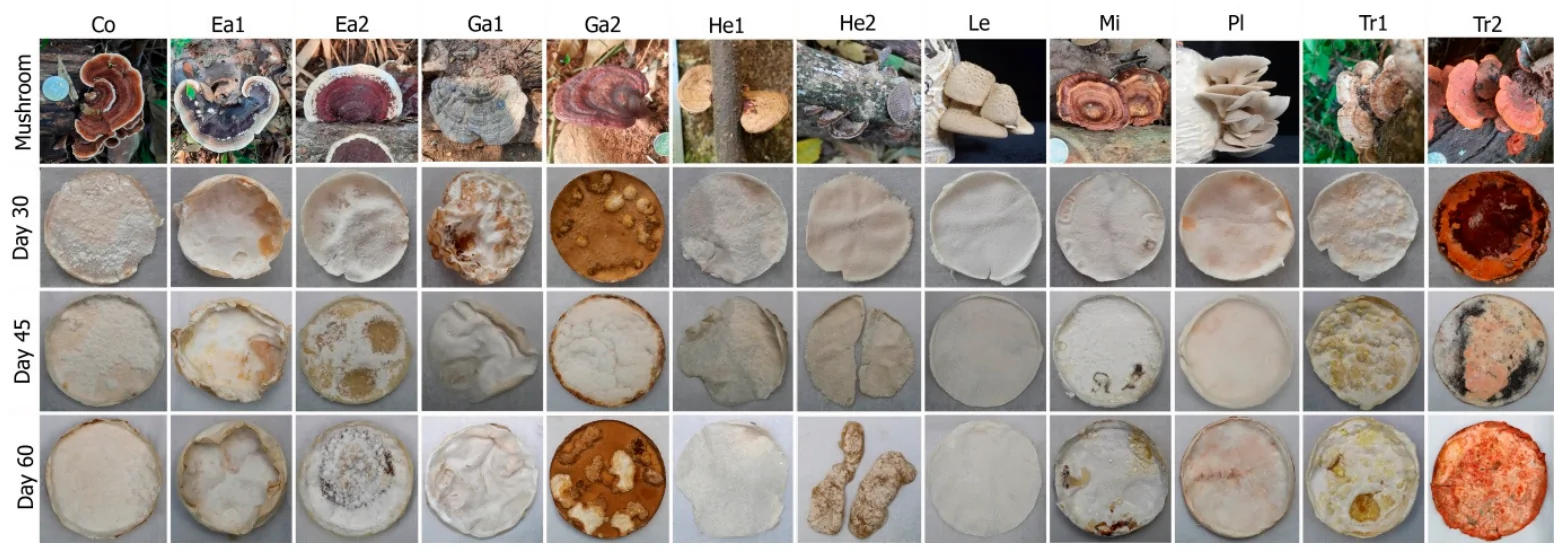
Mycelium mat produced in PDB from selected fungal species on days 30, 45, and 60. (Co) *Coriolopsis brunneoleuca*; (Ea1) *Earliella scabrosa*; (Ea2) *E. scabrosa*; (Ga1) *Ganoderma australe*; (Ga2) *G. tropicum*; (He1) *Hexagonia* sp.; (He2) *Hexagonia* sp.; (Le) *Lentinus squarrosulus*; (Mi) *Microporus xanthopus*; (Pl) *Pleurotus ostreatus*; (Tr1) *Trametes polyzona*; (Tr2) *T. sanguinea*.

The morphology and surface characteristics of the mycelial mats grown on PDB varied among fungal strains (Figure 8, Table 4). With the increase in the incubation period, the mycelium mats were thicker, more robust, and showed no tearing, except for *Hexagonia* sp. (He2). Commercial strain *Pleurotus ostreatus* resulted in better performance than *Lentinus squarrosulus* in the thickness and fluffiness of the mycelium mat. Among wild strains, *Earliella scabrosa* (Ea1), *Ganoderma tropicum*, and *Trametes polyzona* exhibited desirable characteristics (thicker, stronger), while both *Hexagonia* sp. resulted as the weakest strains. All harvested mycelium mats were non-tearing except for *Earliella scabrosa* (Ea2), *Hexagonia* sp. (He1, He2) and *T. sanguinea*. All fungal strains had a smooth surface, while *G. australe* had an underside growth. Mycelium mats of *T. sanguinea* showed light orange pigmentation, while *G. tropicum* showed brown pigmentation.

#### 3.3.2. Morphological Features in Malt Extract Broth

**Figure 9:**
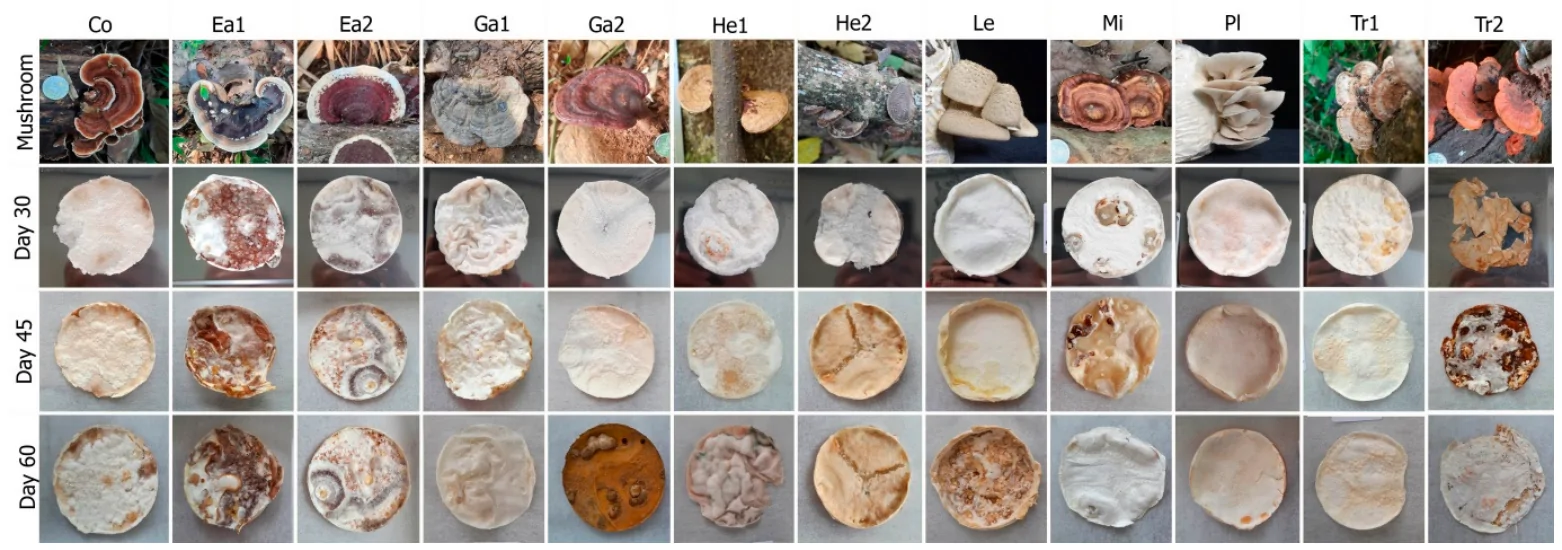
Mycelium mats produced in mushroom mycelium-based biomaterials from selected fungal species on days 30, 45 and 60. (Co) *Coriolopsis brunneoleuca*; (Ea1) *Earliella scabrosa*; (Ea2) *E. scabrosa*; (Ga1) *Ganoderma australe*; (Ga2) *G. tropicum*; (He1) *Hexagonia* sp.; (He2) *Hexagonia* sp.; (Le) *Lentinus squarrosulus*; (Mi) *Microporus xanthopus*; (Pl) *Pleurotus ostreatus*; (Tr1) *Trametes polyzona*; (Tr2) *T. sanguinea*.

The morphology and surface characteristics of the mycelial mats grown on malt extract broth varied according to the fungal strains (Figure 9, Table 5). With a longer incubation period, the mycelium mats were thicker, more robust, and darker in colour. Both commercial strains, *Pleurotus ostreatus* and *Lentinus squarrosulus*, exhibited smooth, fluffy, denser, and rigid mycelium mats. Among wild strains, *Earliella scabrosa* (Ea1) had the thickest and most rigid mycelium mat, while both *Hexagonia* sp. and *Trametes sanguinea* resulted as the weakest strains. Mycelium mats of *T. sanguinea* showed light orange pigmentation. All harvested mycelium mats were non-tearing except for *Hexagonia* sp. (He2) and *T. sanguinea*. All the fungal strains showed smooth mycelium mats, while *E. scabrosa* (Ea1), *G. australe*, and *Hexagonia* sp. (He1) had an undulated appearance. Mycelium mats of *Hexagonia* sp. (He2) and *T. sanguinea* were torn at day 30.

### 3.4. Evaluation of Physical Properties

#### 3.4.1. Moisture Content and Dry Density

The initial moisture content and dry density of twelve fungal strains are shown in Table 6. The initial moisture content ranged from 54.80 to 58.76%, while the dry density varied between 244.21 and 284.05 kg/m^3^. The moisture content was significantly higher in *Coriolopsis brunneoleuca* (Co), with 58.76%, followed by *Pleurotus ostreatus* (Pl). The highest dry density was shown in *Trametes sanguinea* (284.05 kg/m^3^), followed by *Hexagonia* sp. (He2) at 273.87 kg/m^3^. *Hexagonia* sp. (He1) showed significantly higher average shrinkage, with 12.73%, followed by *Lentinus squarrosulus* (Le).

#### 3.4.2. Volumetric Shrinkage

**Figure 10:**
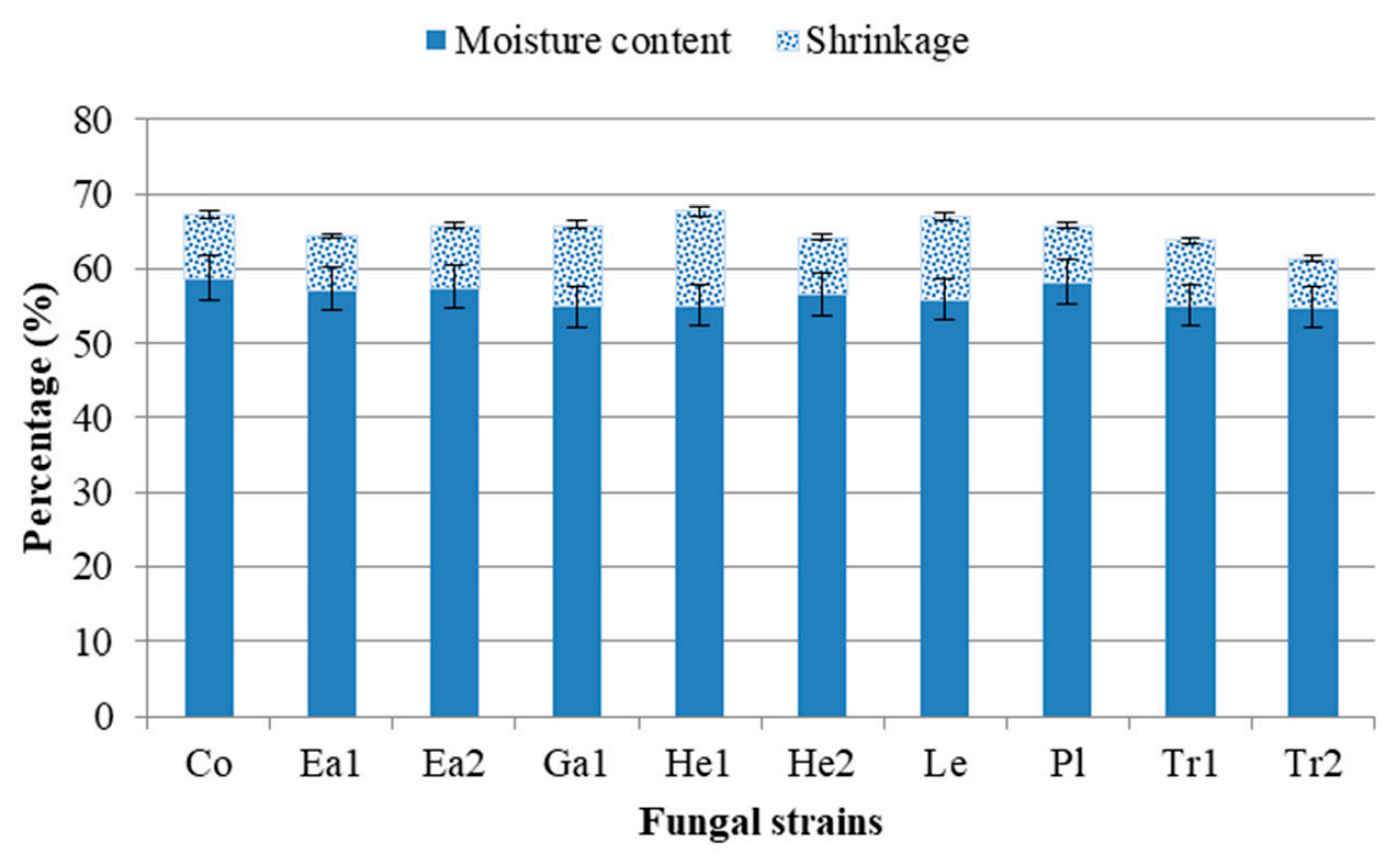
Moisture content and volumetric shrinkage percentage of mushroom mycelium-based biomaterials.

The volumetric shrinkage of the samples ranged between 6.36 and 12.73% (Figure 10, Table 6). The highest shrinkage was observed in *Hexagonia* sp. (He1) at 12.73%, followed by *Lentinus squarrosulus* at 11.16%.

### 3.5. Evaluation of Mechanical Properties

#### 3.5.1. Compression Strength

**Figure 11:**
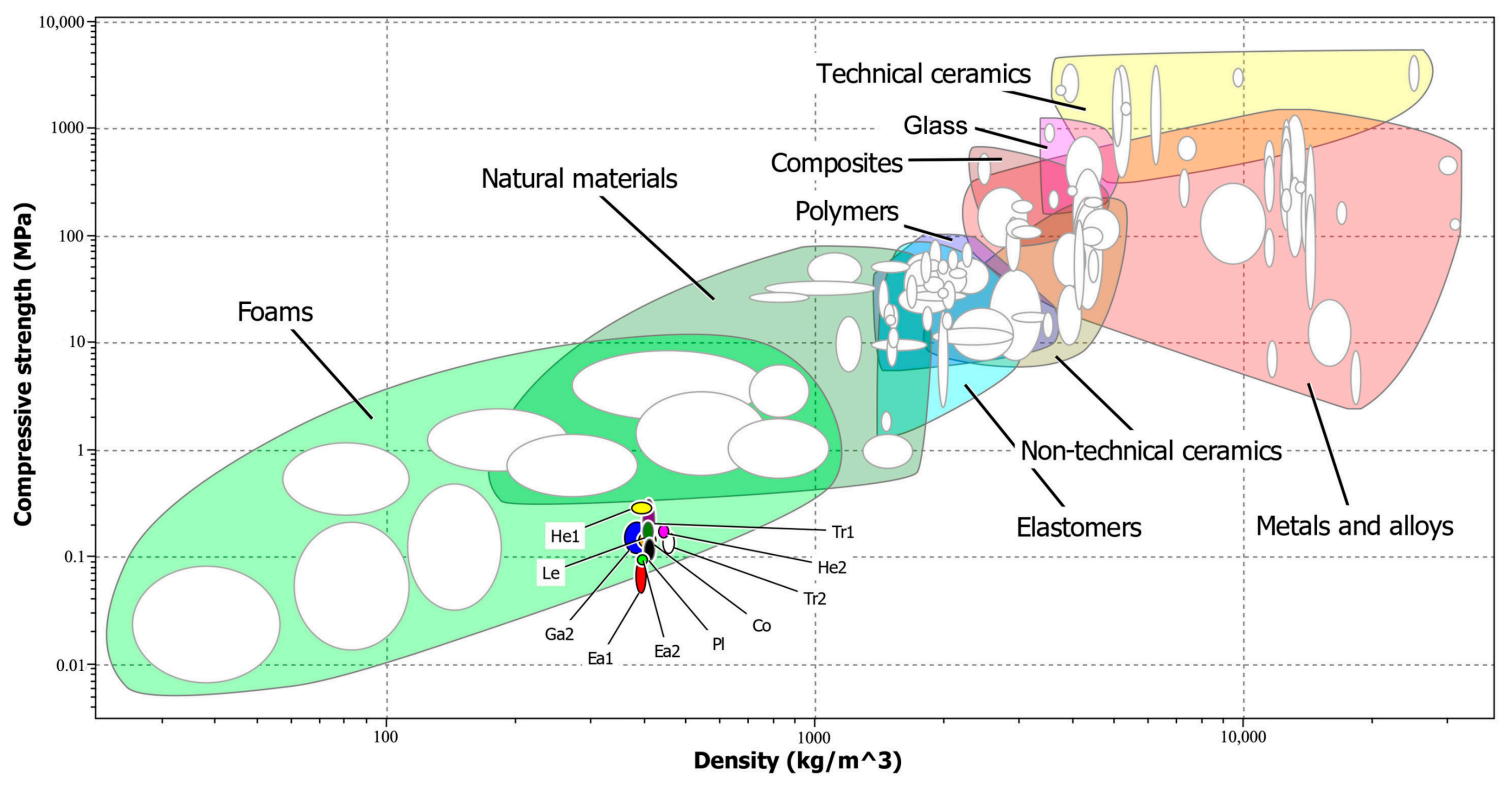
Material property chart (Ashby) comparing mushroom mycelium-based biomaterials from selected fungal strains to typical material families in terms of compressive strength against density. The members of each family are enclosed in envelopes. The image was generated using CES EduPack 2014 (v14.3.5).

The compressive strength of mushroom mycelium-based biomaterial samples derived from the selected fungal species is provided in Table 7. On average, *Hexagonia* sp. (He1) and *Trametes polyzona* exhibited significantly greater compressive strength, with 0.44 MPa and 0.39 MPa, respectively. In contrast, both strains of *Earliella scabrosa* showed the lowest compressive strength at 0.12 MPa. The relationship between compressive strength and density is illustrated using an Ashby chart (Figure 11).

#### 3.5.2. Flexural Strength

**Figure 12:**
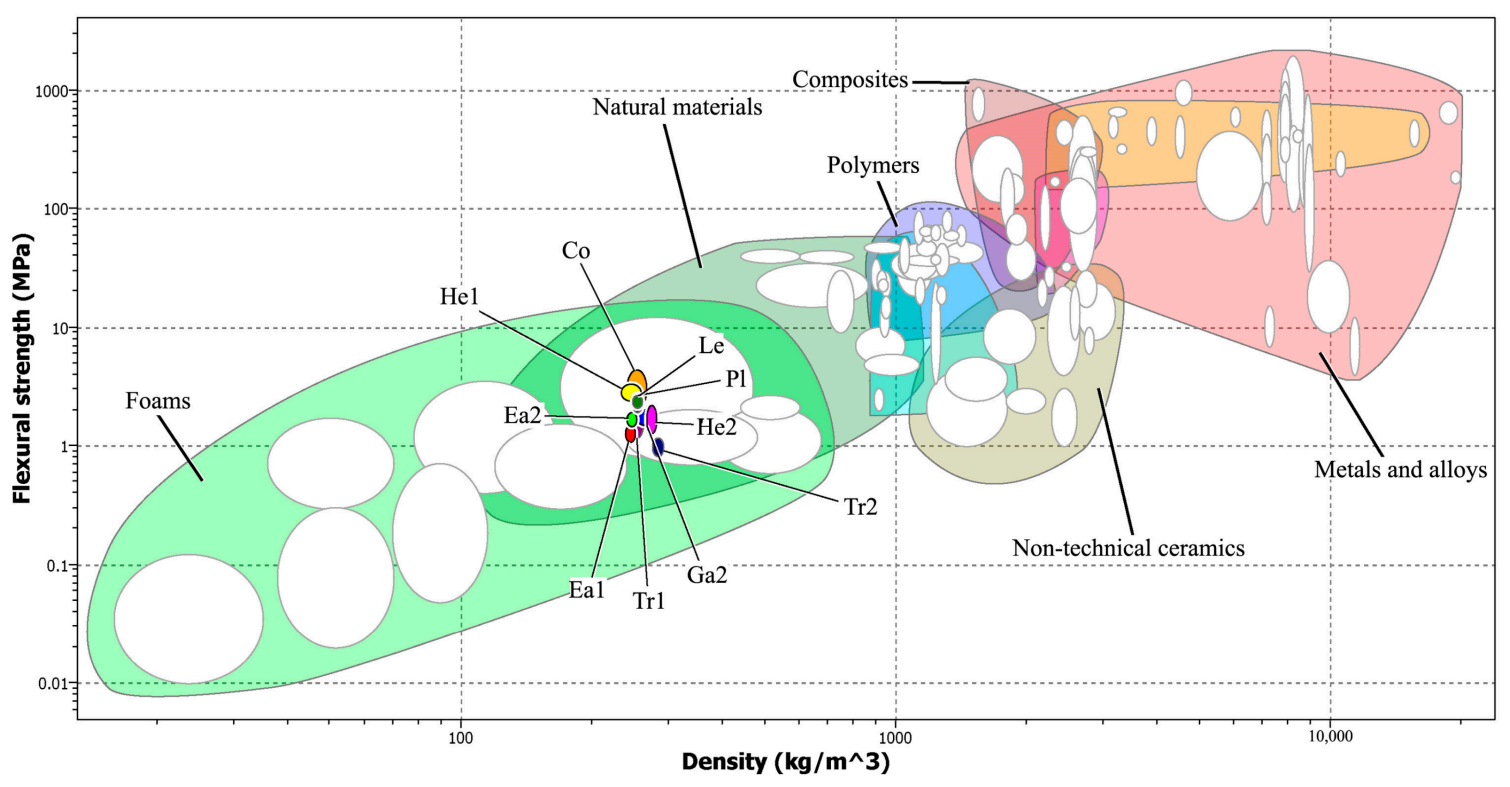
Material property chart (Ashby) comparing mushroom mycelium-based biomaterials from selected fungal strains to typical material families in terms of flexural strength against density. The members of each family are enclosed in envelopes. The chart was generated using CES EduPack 2014 (v14.3.5).

The flexural strength of mushroom mycelium-based biomaterial samples derived from the selected fungal species is provided in Table 7. On average, *Coriolopsis brunneoleuca* and *Hexagonia* sp. (He1) showed the highest flexural strength, with 3.15 MPa and 2.95 MPa, respectively. In contrast, *Trametes sanguinea* showed the lowest flexural strength at 0.95 MPa. The relationship between flexural strength and density is illustrated using an Ashby chart (Figure 12).

#### 3.5.3. Impact Strength

The impact strength of mushroom mycelium-based biomaterial samples from the selected fungal strains is provided in Table 7. On average, *Ganoderma tropicum* showed the highest impact strength (0.38 kJ/m^2^), followed by *Earliella scabrosa* (Ea1) at 0.30 kJ/m^2^. In contrast, *Pleurotus ostreatus* exhibited the lowest impact strength (0.21 kJ/m^2^).

### 3.6. Evaluation of Hydrodynamic Properties

#### 3.6.1. Water Absorption

**Figure 13:**
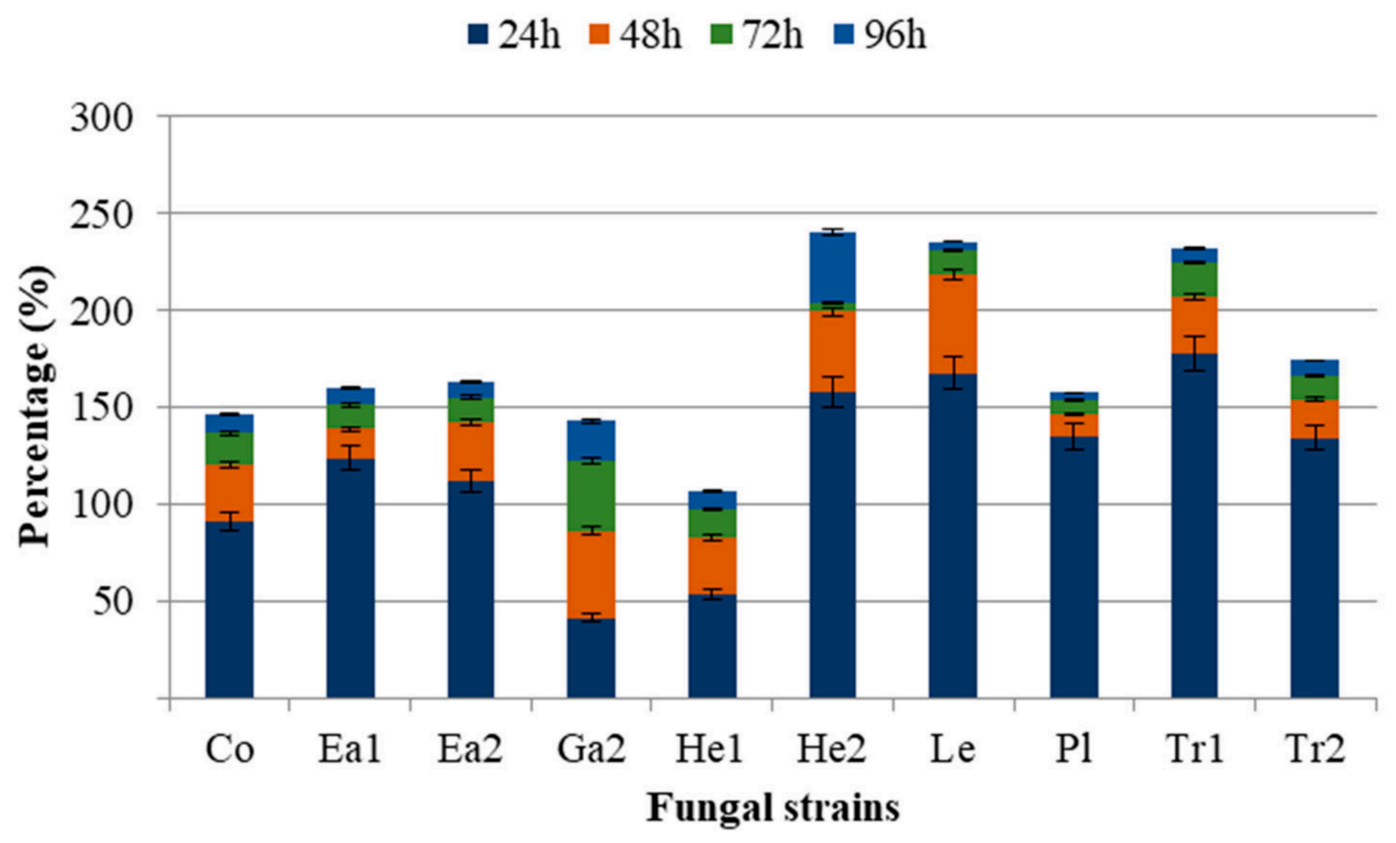
Water absorbance performance of mushroom mycelium-based biomaterials from different fungal strains.

The water absorption capacity of mushroom mycelium-based biomaterial samples derived from different fungal strains is shown in Figure 13. During the initial 24 h period, *Trametes polyzona* exhibited significantly higher water absorption (177%), whereas *Ganoderma tropicum* showed the lowest (41.52%). After a prolonged stage of immersion (24–192 h), water absorption increased substantially, ranging from 107.18% to 240.17%.

#### 3.6.2. Water Contact Angle Analysis

**Figure 14:**
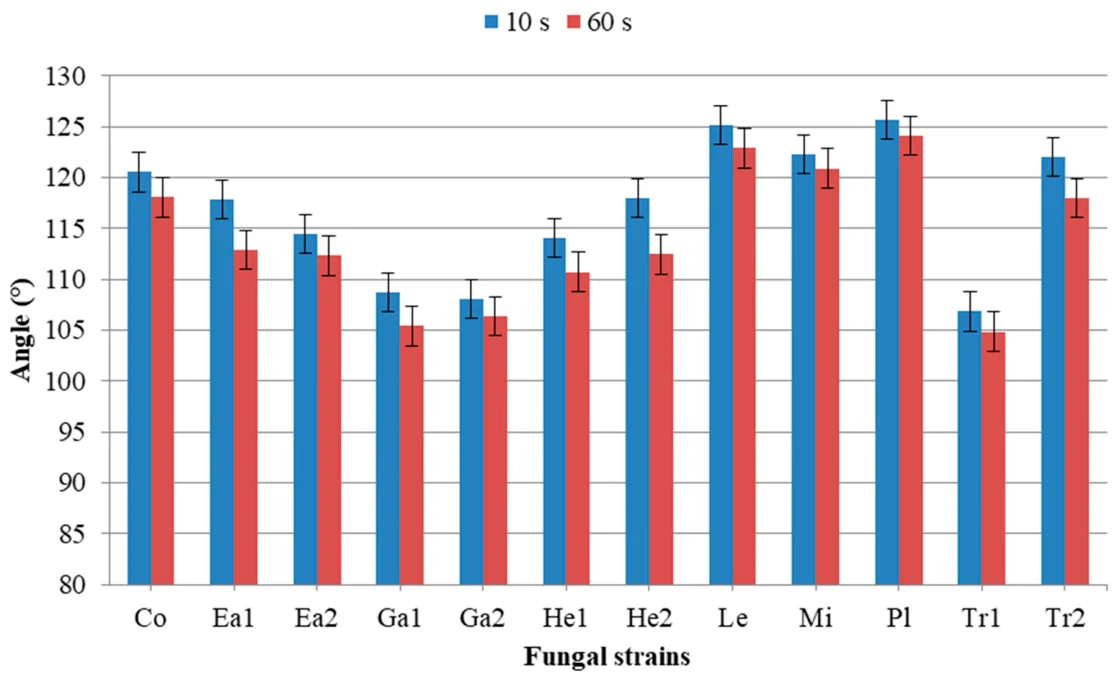
Water contact angle values of pure mycelium mats of twelve mushrooms were measured at 10 s and 60 s.

The water contact angle of pure mycelium mats from twelve fungal strains was evaluated (Figure 14, Table 8). All samples exhibited water contact angle values greater than 90° at 10 s and 60 s, indicating hydrophobic surface characteristics. At 10 s, *Lentinus squarrosulus* and *Pleurotus ostreatus* showed the highest water contact angle values (125.13° and 125.62°), while the lowest value was observed in *Trametes polyzona* (106.82°). At 60 s, *P. ostreatus* maintained the largest WCA (124.11°), followed by *Lentinus squarrosulus* (122.88°). In contrast, *Ganoderma tropicum*, *G. australe*, and *T. polyzona* exhibited comparatively lower water contact angle values at 60 s.

### 3.7. Evaluation of Chemical Properties

**Figure 15:**
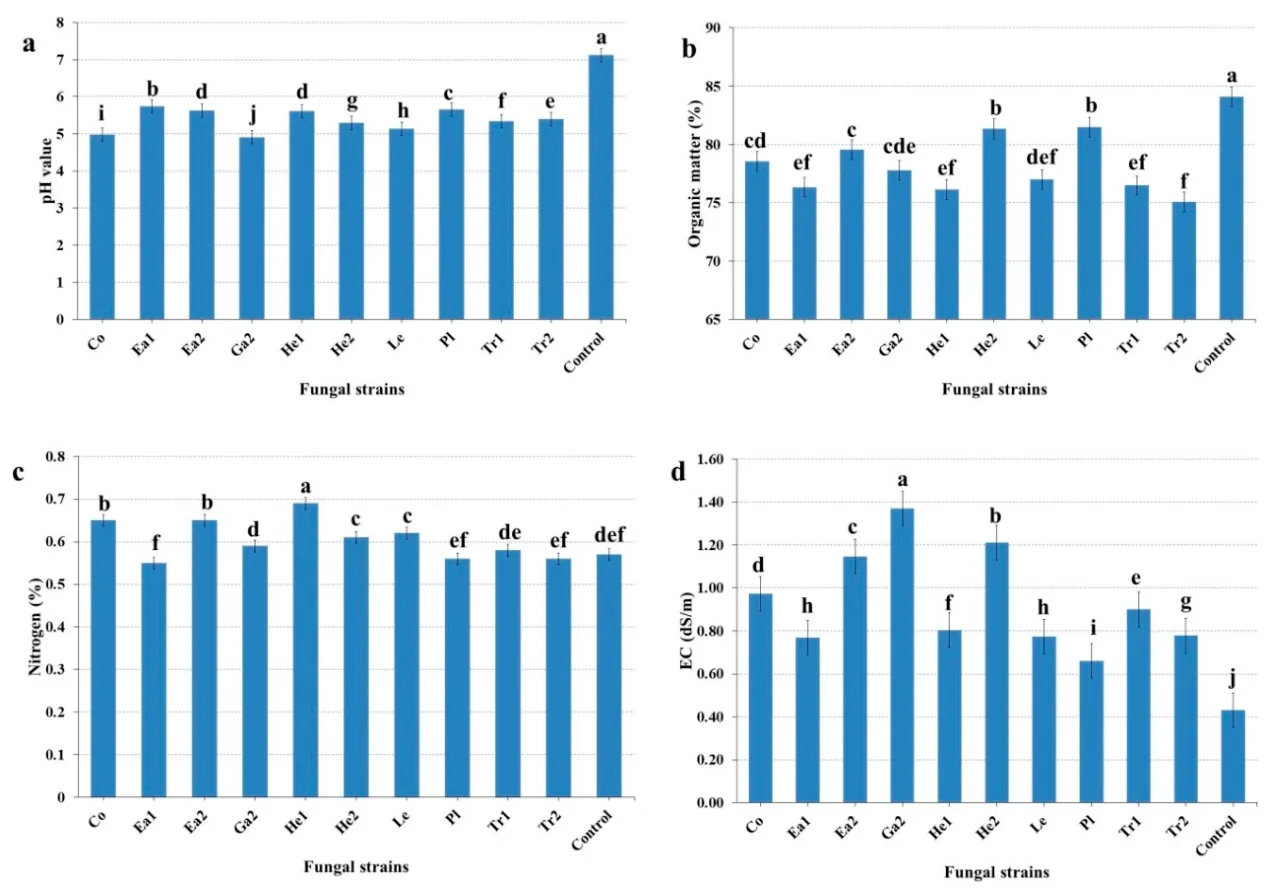
Chemical properties of initial sawdust and test samples produced by fungal strains. (**a**) pH value; (**b**) organic matter content; (**c**) nitrogen content; (**d**) electrical conductivity. Data are presented as means ± standard error. Bars with different letters within individual tests are considered significantly different (*p* < 0.05) according to Duncan’s multiple range tests.

The pH value, organic matter content, nitrogen content and electrical conductivity of test samples and sawdust (control) are shown in Table 9. The initial pH of the growing substrate was 7.11, while the final pH values of the test samples ranged from 4.90 to 5.74 (Figure 15a). Overall, the pH decreased significantly compared to the initial pH value of the sawdust substrates. The organic matter content decreased in all mushroom mycelium-based biomaterials across the fungal strains (Figure 15b). Notably, nitrogen content increased in all the mushroom mycelium-based biomaterials, with increased levels in *Coriolopsis brunneoleuca*, *Earliella scabrosa* (Ea2), *Hexagonia* sp. (He1), *Hexagonia* sp. (He2), and *Lentinus squarrosulus* (Figure 15c). Electrical conductivity of test samples ranged from 0.66 to 1.37 dS/m and was significantly higher than sawdust alone (0.43 dS/m) (Figure 15d).

### 3.8. Scanning Electron Microscopy

**Figure 16:**
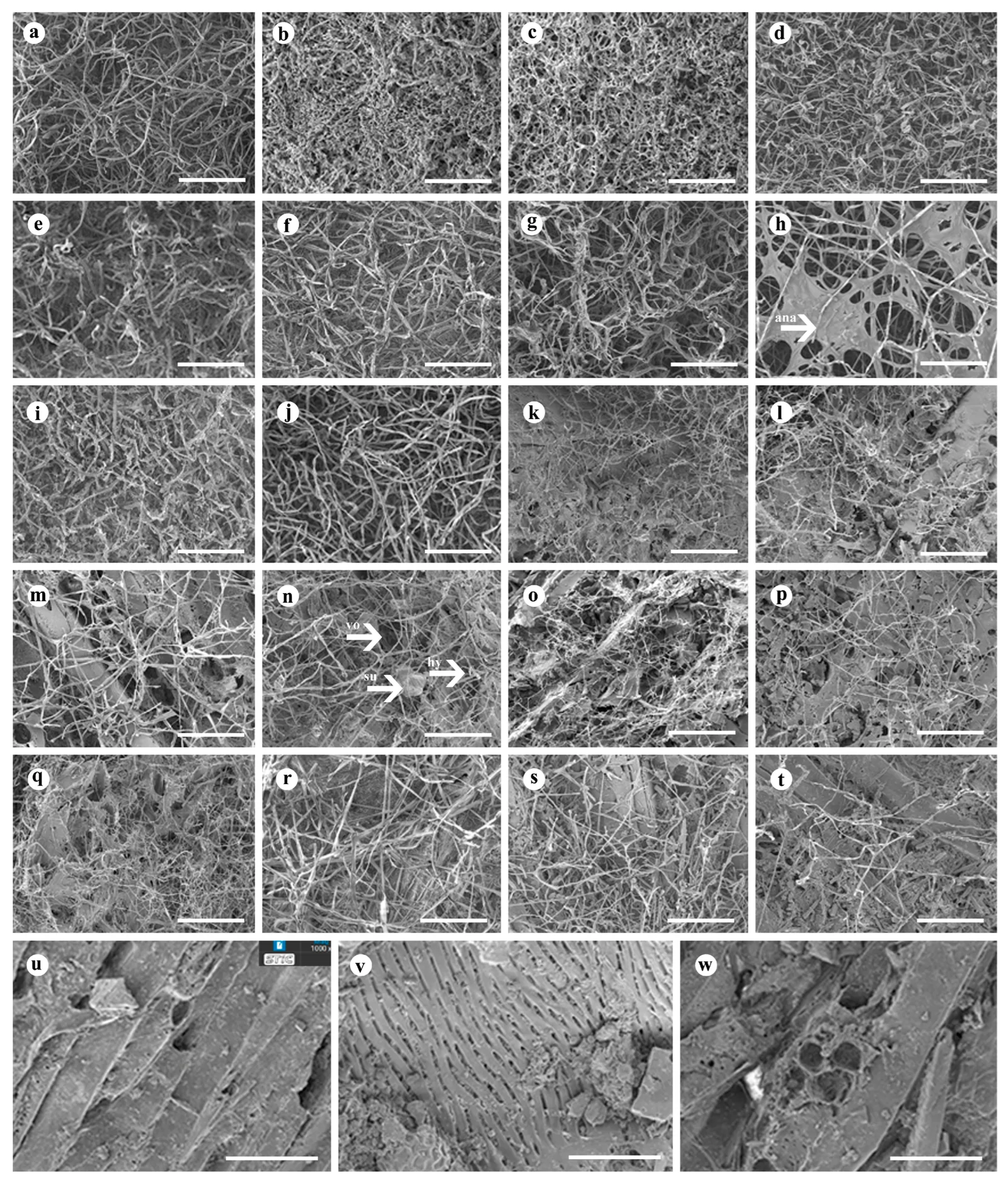
Scanning electron microscopy images of the test samples obtained in this study. Surface morphology of test samples developed from (**a**) *Coriolopsis brunneoleuca*; (**b**,**c**) *Earliella scabrosa*; (**d**) *Ganoderma tropicum*; (**e**,**f**) *Hexagonia* sp.; (**g**) *Lentinus squarrosulus*; (**h**) *Pleurotus ostreatus*; (**i**) *Trametes polyzona*; (**j**) *T. sanguinea*. Cross-section views of samples developed from (**k**) *Coriolopsis brunneoleuca*; (**l**,**m**) *Earliella scabrosa*; (**n**) *Ganoderma tropicum*; (**o**,**p**) *Hexagonia* sp.; (**q**) *Lentinus squarrosulus*; (**r**) *Pleurotus ostreatus*; (**s**) *Trametes polyzona*; (**t**) *T. sanguinea*. Uncolonised sawdust (**u**–**w**). ana = anastomosis hypha, hy = hypha, su = substrate, vo = voids. Scale bars: a−w = 50 μm.

The hyphal structures of selected fungal species grown in sawdust were examined using a scanning electron microscope, as shown in Figure 16. The surface view shows a dense hyphal network grown on the surface rather than within the interior. The hyphae are either as fibrillar structures or as anastomosis (fused) hyphae, with the latter being more prominent in *Pleurotus ostreatus*. In some cases, the hyphal filaments appeared flattened. Visual inspection indicated that the surface mycelium of *Earliella scabrosa* (Figure 16b,c) was considerably denser compared to other fungal species, which exhibited a more loosely interwoven and fluffy structure. Cross-sectional views showed the arrangement of hyphae, substrates, and voids, where multidimensional networks bind discrete substrate particles, contributing to structural rigidity. In contrast, microstructure visualisation of the sawdust substrate (control) revealed a rough surface composed of discrete particles of varying size without hyphal growth.

### 3.9. Thermogravimetric Analysis on Pure Mycelium Mats

The thermal behaviour of pure mycelium mats from selected fungal species was evaluated using thermogravimetric analysis (TGA) and derivative thermogravimetric (DTGA) curves (Figure S1). All samples exhibited similar degradation patterns consisting of three main stages. The first event was observed below 100 °C and corresponded to moisture evaporation, resulting in approximately 8% mass loss. The second event represented the primary thermal degradation phase, with initial degradation temperature ranging from approximately 285 °C for *Coriolopsis brunneoleuca* to 306 °C for *Hexagonia* sp. (He1), resulting in approximately 63% mass loss. The third event, observed around 810 °C, was associated with the decomposition of non-degraded waste materials and impurities, contributing approximately 20% mass loss. Among the samples, the lowest moisture content was recorded in *Ganoderma australe* (6.78%), while the highest was observed in *G. tropicum* (8.86%). Additionally, the lowest initial degradation temperature (±285 °C) was found in *Coriolopsis brunneoleuca*, whereas the highest (±306 °C) was observed in *Hexagonia* sp. (He1).

### 3.10. Fourier Transform Infrared Spectroscopy

Fourier transform infrared spectroscopy spectra of pure mycelium of the selected fungal strains, along with their characteristic absorption bands, are shown in Figure S2. Chemically significant regions identified in the spectra include hydroxyl groups (3200–3400 cm^−1^), C–H stretching vibrations in the fatty acid region (2800–3000 cm^−1^), protein-associated bands dominated by C=O (amide I) and N–H bending (amide II) (1500–1700 cm^−1^), and polysaccharide region (900–1200 cm^−1^). Detailed peaks in the obtained spectra are provided in Table S1.

### 3.11. Soil Burial Biodegradability

**Figure 17:**
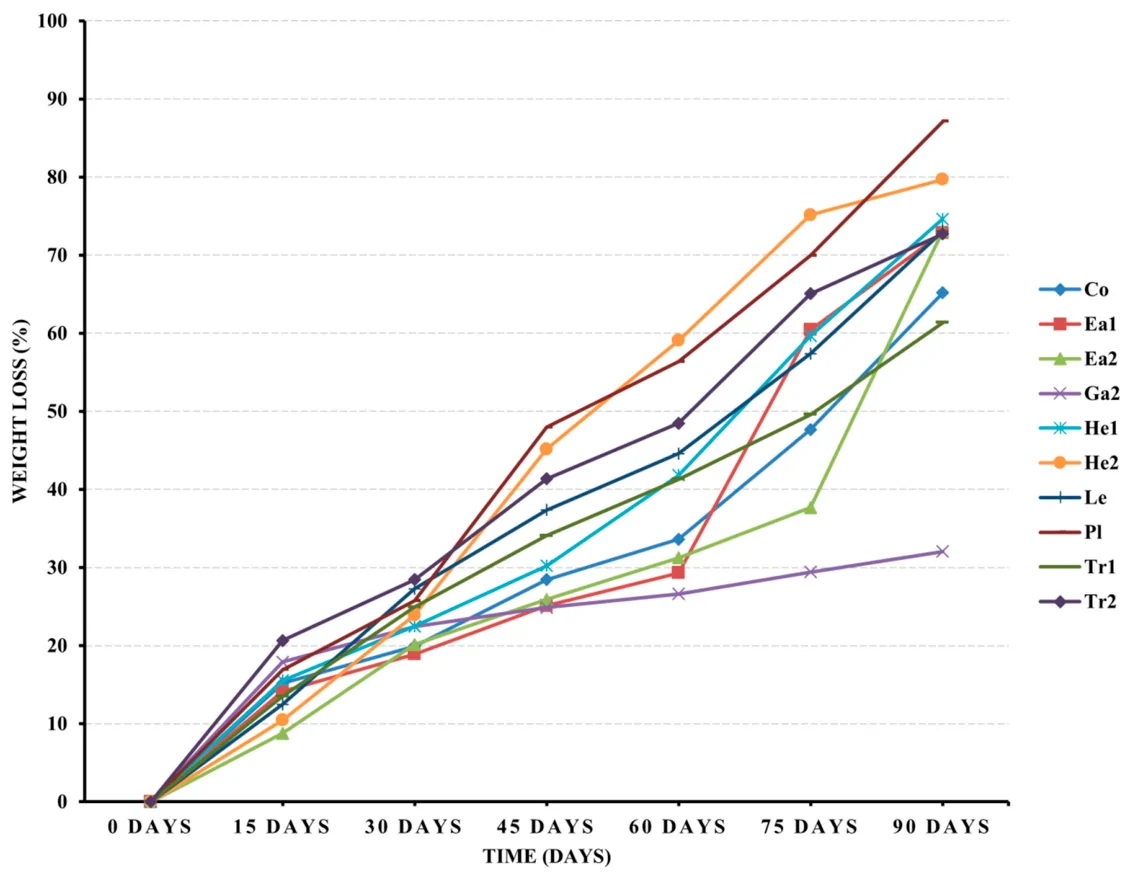
Cumulative percentage weight loss of mushroom mycelium-based biomaterials during the soil burial degradability test.

**Figure 18:**
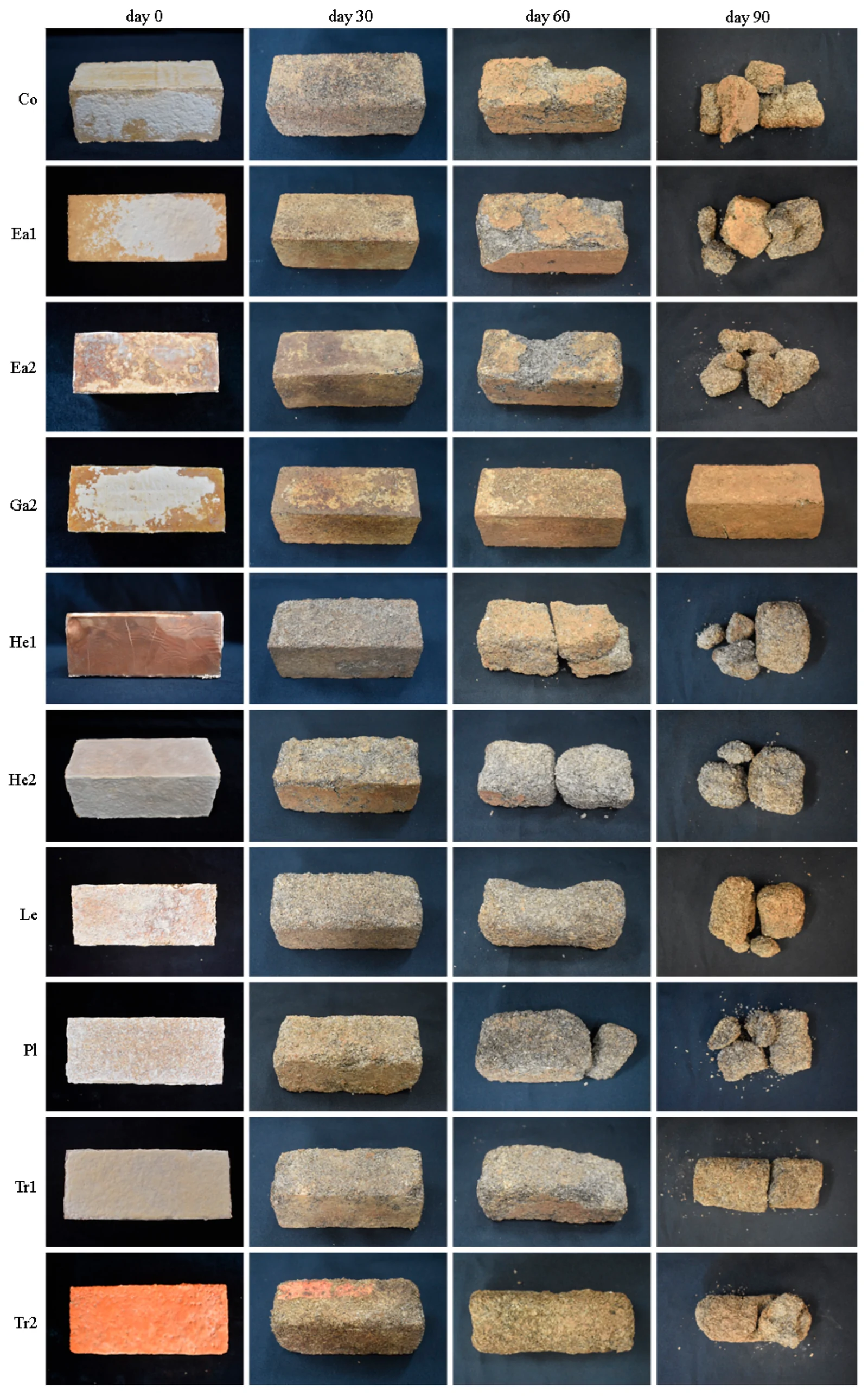
Visual appearance of mushroom mycelium-based biomaterials from selected fungal species at 0, 30, 60, and 90 days during the soil burial degradability test.

All the test samples of ten fungal strains gradually disintegrate over time, except those derived from *Ganoderma tropicum*. The cumulative weight loss of all samples exceeded 60% after 90 days of soil burial except *G. tropicum*, with only 32% weight loss (Figure 17, Table 10). Among the tested samples, *Pleurotus ostreatus* exhibited the highest cumulative weight loss (87.11%), followed by *Hexagonia* sp. (He2) at 79.70%. The visual appearance of the samples at 0, 30, 60, and 90 days of soil burial is shown in Figure 18. During the first 30 days, surface mycelial mats showed signs of erosion, although the overall shape and size remained unchanged. By day 60, the samples were either highly eroded and/or fragmented. After day 90, most samples had broken into smaller pieces. However, *G. tropicum* retained its original shape and showed no disintegration compared to the other samples. Additionally, the samples exhibited a gradual darkening and became blackish with increasing duration of soil burial.

### 3.12. Viability Maintenance of Fungal Strains

**Figure 19:**
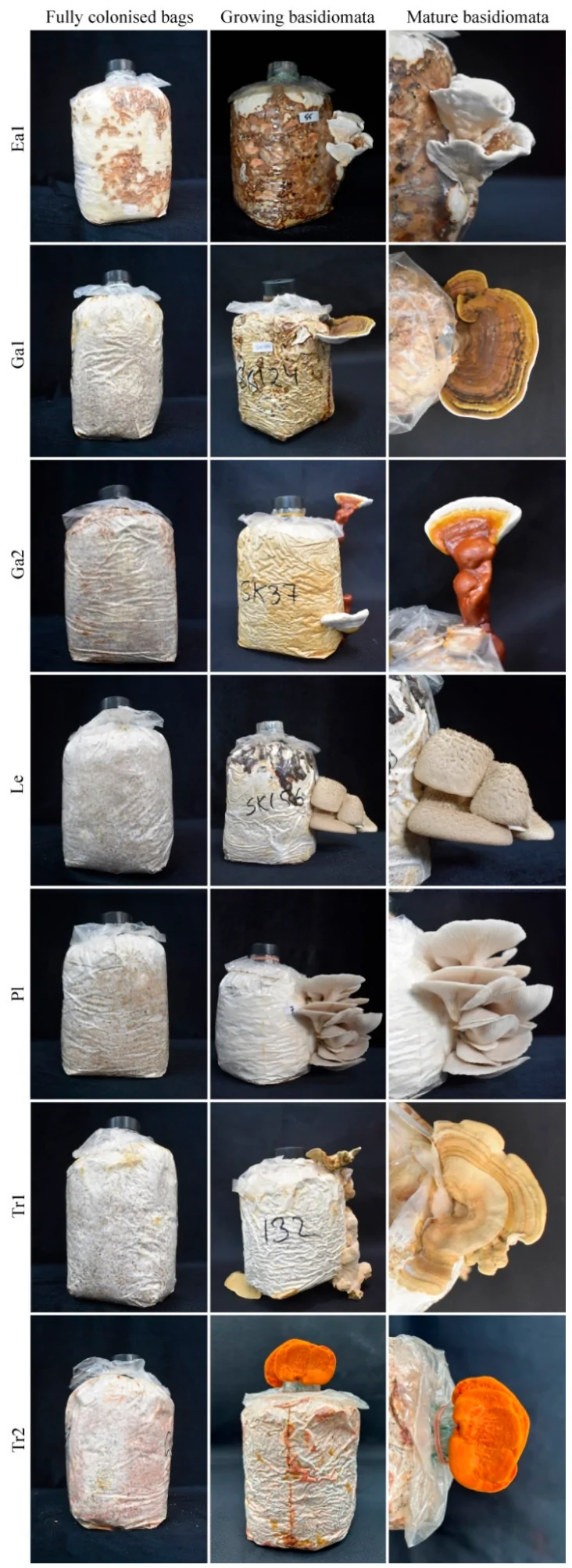
Viability maintenance of mushroom strains showing fully colonised substrate bags, development of basidiomata within the bags, and mature basidiomata.

Substrate bags fully colonised by the mycelia of the selected fungal species and incubated in the fruiting chamber successfully produced basidiomata (Figure 19). Mature basidiomata were harvested, and fresh tissue was re-isolated to obtain pure cultures for subsequent studies.

### 3.13. Prototype Development

**Figure 20:**
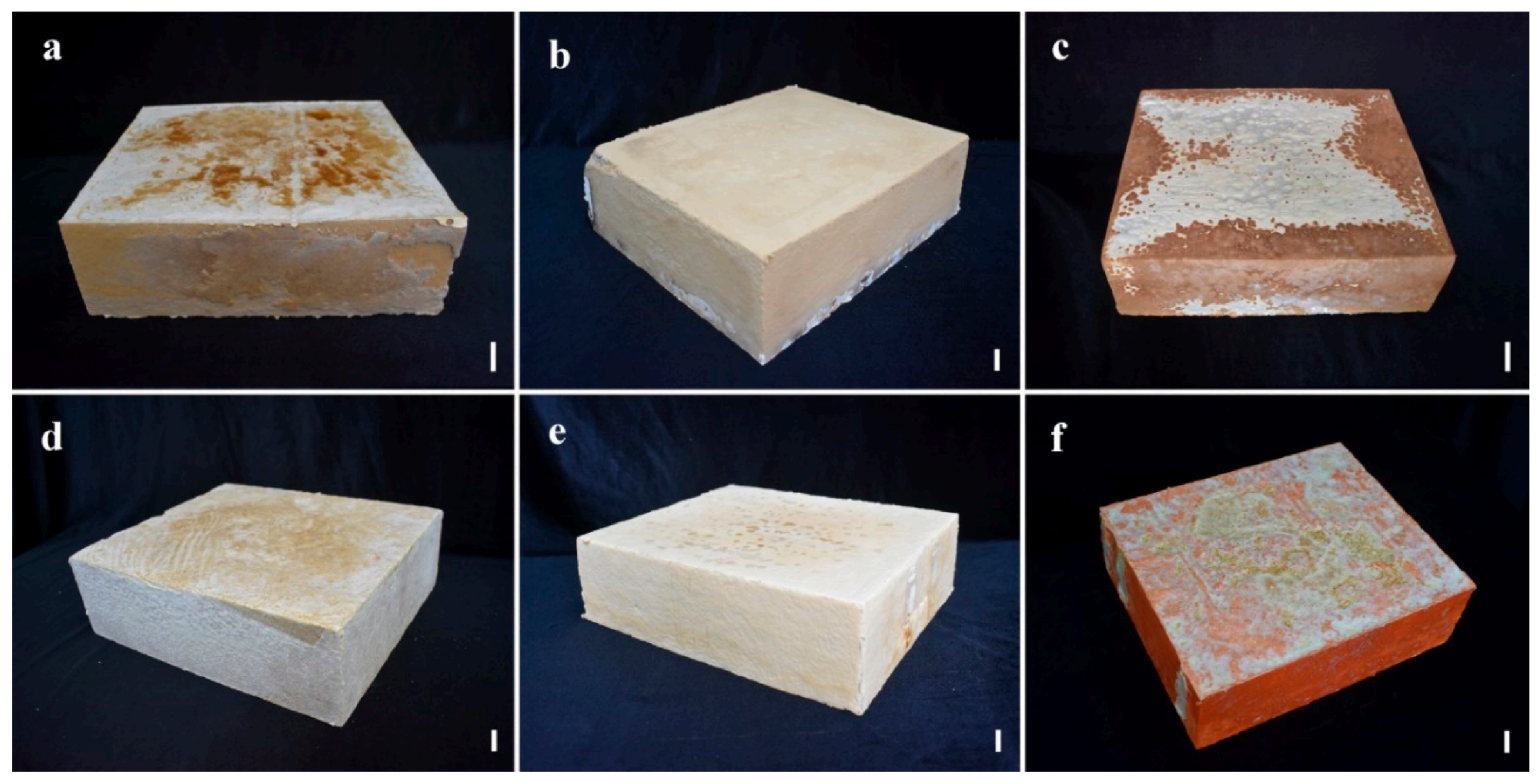
Representative individual blocks of prototypic wall materials developed using selected fungal species and sawdust substrates. (**a**) *Earliella scabrosa* (Ea1); (**b**) *E. scabrosa* (Ea2); (**c**) *Hexagonia* sp. (He1); (**d**) *Pleurotus ostreatus* (Pl); (**e**) *Trametes polyzona* (Tr1); (**f**) *T. sanguinea* (Tr2). Scale bars: a–f = 2 cm.

Individual cuboid blocks with dimensions of 30 × 25 × 10 cm were fabricated and subsequently deactivated (Figure 20). The blocks exhibited variations in surface colour and texture that correspond to the different fungal species used. The final products showed acceptable geometric dimensions, structural stability, and rigidity and were free from contamination by other fungal species.

## 4. Discussion

Accurate identification of fungal specimens, based on an integrated approach combining macromorphological and ITS-based phylogenetic analyses, provides the scientific basis for their application in mushroom mycelium-based biomaterials. Variations in mycelial growth rate and density within the same culture medium indicate species-specific substrate preferences. The volumetric shrinkage of mushroom mycelium-based biomaterial samples observed in this study is consistent with a previous study (3.14–16.31%) [24], indicating acceptable dimensional stability for construction, insulation, and interior design blocks.

Analysis of the Ashby charts reveals that the mechanical properties of mushroom mycelium-based biomaterial samples (compressive strength vs. density and flexural strength vs. density) fall within the range of natural materials. The investigation of the mushroom mycelium-based biomaterial samples in this study results in mechanical properties appropriate for use as a foam substitute. Notably, the observed differences in compressive strength among fungal species may be attributable to variations in the mycelial network and density. Furthermore, the highest and lowest impact strength observed in *Ganoderma tropicum* and *Pleurotus ostreatus* might be attributed to their trimitic and monomitic hyphal systems, respectively. These findings provide a basis for establishing selection criteria for fungal species in mushroom mycelium-based biomaterial development.

Water absorption, up to 177% in 24 h, is one the limitations of mushroom mycelium-based biomaterials that exceeds synthetic materials [8,17,19]. This suggests the use of the material is preferably for indoor purposes or dry areas. The water contact angle values reflect the hydrophobic nature of pure mycelium mats, citing the values above 90°, and support the results of previous studies [8,25]. This behaviour indicates the potential application of mushroom mycelium in several sectors, including packaging and medical scaffolds [20,26]. The proteins, such as mannoproteins and hydrophobins, present on the outermost layer of the fungal cell wall might be responsible for the hydrophobic nature of mushroom mycelium-based biomaterials [27,28].

Regarding the chemical analyses, the results are in accordance with prior studies [6,8,19,29]. The decrease in pH and organic matter content in mushroom mycelium-based biomaterial samples resulted from enzymatic digestion of substrates by the mycelium and subsequent leaching [22,29].

SEM analysis revealed a microstructural arrangement of dense, interwoven hyphal networks within mushroom mycelium-based biomaterial samples. The multidimensional mycelium network acted as a natural adhesive, providing shape stability and enhanced mechanical strength to the final materials. The denser hyphal mass on the sample surface, compared with the internal part, is associated with oxygen availability [30]. The anaerobic environment and higher temperature occurring in samples with dense mycelium layers are unfavourable conditions for colonisation [31]. However, increasing incubation time could promote colonisation of the inner part of products [32,33].

The mycelium surface layer of mushroom mycelium-based biomaterials affects its hydrophobicity behaviour. The differences in void material among fungal strains are attributable to their hyphal networks. The voids and spongy mycelial network help absorb external pressure shock, thereby featuring suitability for packaging purposes. Additionally, the flattened hyphae, under heat treatment, were due to the failure of hyphae to maintain internal hydrostatic pressure [25,30]. Regarding sawdust, its surface roughness provides increased surface area and microcracks or voids, which facilitate hyphal penetration, fermentation, and nutrient absorption.

In thermal analysis, the weight loss of the sample at lower temperatures is attributed to the removal of free and chemically linked water [34,35,36]. The weight loss in the second stage is attributed to the thermal degradation of lignocellulosic components (i.e., cellulose, hemicellulose and lignin) occurring between 220 and 410 °C, thereby exhibiting thermal stability [35,37,38,39]. The proportion of impurities or char contributes to flame resistivity. FTIR spectroscopy revealed chemically bonded key functional groups: hydroxyl, lipid, protein, and polysaccharides, with mostly similar signals for all fungal species. Samples from *Microporus xanthopus* showed stronger signals in some of the abovementioned functional groups.

The soil burial degradability test provides strong evidence that mushroom mycelium-based biomaterials are completely biodegradable within a short period in active soil. Microbial enzymes might have helped decompose and disintegrate mushroom mycelium-based biomaterials over time, thereby leaving no end product waste in the environment [8]. Heavy decomposition of the samples from *Pleurotus ostreatus* might be attributed to a monomitic hyphal system, while *Ganoderma tropicum* may take somewhat longer to decompose due to its trimitic hyphal system. Notably, biological decomposition leads to balanced nutrient cycling in situ.

Mushroom mycelium-based biomaterial production faces the culture degeneration issue due to repeated subculturing [40,41]. However, this study shows that mature basidiomata obtained through the cultivation technique could be used for re-isolation to continue mushroom mycelium-based biomaterial production. Additionally, successfully developed building blocks, using the abovementioned fungal species and rubber sawdust, provide insights for commercial production. Additionally, various construction, household, and decorative items, including vases, bowls, and blocks, were designed and fabricated. This practical output is also attributed to multidisciplinary characteristics as described above.

In summary, this study accounts for the first comprehensive explanation of the use of *Coriolopsis brunneoleuca*, *Ganoderma tropicum*, *Hexagonia* sp., *Microporus xanthopus*, *Trametes polyzona*, and *T. sanguinea* in mushroom mycelium-based biomaterial development and characterisation. Additionally, the successful development of these prototypes provides key insights for indoor applications, such as building blocks, insulating walls, and packaging [8]. Therefore, this study broadens the range of potential mushroom species in fulfilling basic material properties for wider applications.

## 5. Conclusions

In this study, 10 wild mushrooms and two commercial edible fungal strains were used to prepare mushroom mycelium-based biomaterial test samples. The mycelial growth performance in agar media (PDA and MEA) and in sawdust could serve as preliminary criteria for understanding culture characteristics. Pure mycelium mats were successfully developed in broth media (PDB and MEB), thereby providing a basis for further exploration to strengthen them. The mushroom mycelium-based biomaterial test samples exhibited suitable physical and mechanical behaviour comparable to synthetic foams, suggesting it as an alternative material. The fungal strains showed thermal stability, leaving more than 30% of residue at around 300 °C. Additionally, mycelium showed higher hydrophobicity, with all results above 105° both at 10 and 60 s. Most of the fungal strains showed a higher percentage of weight loss (above 60%) at around 90 days of soil burial, providing insights into their sustainability and biodegradability. The prototypic mushroom mycelium-based biomaterials indicate the scope of scale-up, focusing on packaging, insulation, and the non-load-bearing construction sector.

## Figures and Tables

**Table 1:** Details of selected fungal strains used in this study, with voucher number, family, hyphal system, collected area, and forest type.

Fungal Strains	Vouchers	Family	Hyphal System	Collected Area (Province)	Source
*Coriolopsis brunneoleuca* (Co)	MFLUCC 24-0265	Polyporaceae	Trimitic	Chiang Mai	Mixed deciduous forest
*Earliella scabrosa* (Ea1)	MFLUCC 24-0263	Polyporaceae	Trimitic	Chiang Rai	Mixed deciduous forest
*E. scabrosa* (Ea2)	MFLUCC 24-0268	Polyporaceae	Trimitic	Chiang Rai	Mixed deciduous forest
*Ganoderma australe* (Ga1)	MFLUCC 24-0262	Polyporaceae	Trimitic	Chiang Rai	Mixed deciduous forest
*G. tropicum* (Ga2)	MFLUCC 24-0267	Polyporaceae	Trimitic	Chiang Rai	Mixed deciduous forest
*Hexagonia* sp. (He1)	MFLUCC 24-0266	Polyporaceae	Trimitic	Chiang Mai	Mixed deciduous forest
*H.* sp. (He2)	MFLUCC 24-0270	Polyporaceae	Trimitic	Chiang Mai	Mixed deciduous forest
*Lentinus squarrosulus* (Le)	MFLUCC 24-0271	Polyporaceae	Dimitic	Chiang Rai	Local market
*Microporus xanthopus* (Mi)	MFLUCC 24-0261	Polyporaceae	Trimitic	Chiang Rai	Mixed deciduous forest
*Pleurotus ostreatus* (Pl)	MFLUCC 24-0260	Pleurotaceae	Monomitic	Chiang Rai	Local market
*Trametes polyzona* (Tr1)	MFLUCC 24-0269	Polyporaceae	Trimitic	Chiang Rai	Mixed deciduous forest
*T. sanguinea* (Tr2)	MFLUCC 24-0264	Polyporaceae	Trimitic	Chiang Rai	Mixed deciduous forest

**Table 2:** Average growth rate and mycelial density of the selected fungal strains in PDA and MEA media.

Fungal Strains	Average Growth Rate (mm/day)				Mycelial Density on Day 14	
	PDA		MEA		PDA	MEA
	95% Confidence Interval	Average	95% Confidence Interval	Average		
*Coriolopsis brunneoleuca* (Co)	10.09, 12.51	11.30 ± 0.97 ^bc^	10.18, 13.56	11.87 ± 1.36 ^bcd^	3+	3+
*Earliella scabrosa* (Ea1)	10.38, 11.86	11.12 ± 0.60 ^bc^	7.24, 13.59	10.42 ± 2.56 ^de^	5+	5+
*E. scabrosa* (Ea2)	8.13, 8.60	8.36 ± 0.19 ^d^	8.91, 9.73	9.32 ± 0.33 ^e^	5+	5+
*Ganoderma australe* (Ga1)	8.25, 12.95	10.60 ± 1.89 ^c^	8.08, 12.88	10.48 ± 1.93 ^de^	3+	3+
*G. tropicum* (Ga2)	n/a *	n/a *	n/a *	n/a *	n/a *	n/a *
*Hexagonia* sp. (He1)	8.98, 12.42	10.70 ± 1.39 ^c^	8.61, 14.59	11.60 ± 2.41 ^cde^	5+	5+
*Hexagonia* sp. (He2)	3.35, 5.12	4.24 ± 0.72 ^e^	3.59, 7.81	5.70 ± 1.70 ^f^	2+	3+
*Lentinus squarrosulus* (Le)	11.91, 14.22	13.07 ± 0.93 ^a^	12.96, 15.21	14.08 ± 0.90 ^ab^	2+	3+
*Microporus xanthopus* (Mi)	11.60, 13.94	12.77 ± 0.94 ^ab^	7.01, 13.72	10.37 ± 2.70 ^de^	4+	4+
*Pleurotus ostreatus* (Pl)	9.17, 11.03	10.10 ± 0.75 ^c^	10.87, 12.33	11.60 ± 0.59 ^cde^	5+	5+
*Trametes polyzona* (Tr1)	9.61, 15.59	12.60 ± 2.40 ^ab^	13.30, 15.14	14.22 ± 0.74 ^a^	4+	4+
*T. sanguinea* (Tr2)	12.04, 15.12	13.58 ± 1.24 ^a^	11.62, 14.14	12.88 ± 1.01 ^abc^	3+	3+

Note: Values are represented as mean ± standard deviation. Within a column, values followed by the same letter are not significantly different (*p* < 0.05) by Duncan’s multiple range test. * Specimen failed to grow.

**Table 3:** Mycelial growth rate and mycelial characteristics of selected fungal strains in lignocellulosic substrate.

Fungal Strains	Growth Rate (mm/day)		Mycelial Characteristics
	95% Confidence Interval	Average	
*Coriolopsis brunneoleuca* (Co)	2.62, 3.08	3.09 ± 0.38 ^g^	white, mycelium thick at periphery, thin at centre, 3+
*Earliella scabrosa* (Ea1)	3.36, 5.67	4.51 ± 0.93 ^cde^	white, concentric mycelium pattern, 3+
*E. scabrosa* (Ea2)	3.42, 5.40	4.41 ± 0.80 ^cde^	white, concentric mycelium pattern, 4+
*Ganoderma australe* (Ga1)	6.02, 6.62	6.32 ± 0.24 ^a^	white, uniform mycelium in the entire plate, 3+
*G. tropicum* (Ga2)	5.53, 6.56	6.04 ± 0.41 ^a^	white, uniform mycelium in the entire plate, 3+
*Hexagonia* sp. (He1)	2.90, 4.21	3.55 ± 0.53 ^fg^	white, concentric ring of mycelium, 4+
*Hexagonia* sp. (He2)	3.36, 4.93	4.14 ± 0.63 ^def^	white, uniform mycelium in the entire plate, 4+
*Lentinus squarrosulus* (Le)	4.61, 6.55	5.58 ± 0.78 ^ab^	white, uniform mycelium in the entire plate, 2+
*Microporus xanthopus* (Mi)	3.32, 4.11	3.71 ± 0.32 ^efg^	white, uniform mycelium in the entire plate, 3+
*Pleurotus ostreatus* (Pl)	4.28, 5.09	4.69 ± 0.32 ^cd^	white, mycelium thick at periphery, thin at centre, 4+
*Trametes polyzona* (Tr1)	4.41, 5.93	5.17 ± 0.61 ^bc^	greyish white, uniform mycelium in the entire plate, 3+
*T. sanguinea* (Tr2)	3.59, 5.58	4.58 ± 0.80 ^cd^	orangish white, uniform mycelium in entire plate, 3+

Note: Values are represented as mean ± standard deviation. Within each column, values followed by the same letter are not significantly different (*p* < 0.05) by Duncan’s multiple range test.

**Table 4:** Morphological features of pure mycelium mats of selected fungal strains grown in potato dextrose broth.

Fungal Strains	Colour	Rigidity	Mycelial Density	Surface Texture
*Coriolopsis brunneoleuca* (Co)	Gray	No tearing	3+	Smooth, fluffy
*Earliella scabrosa* (Ea1)	White, brown pigmentation	No tearing	4+	Smooth
*E. scabrosa* (Ea2)	White, brown pigmentation	Tearing on day 30, no tearing later	3+	Smooth
*Ganoderma australe* (Ga1)	White, brown pigmentation	No tearing	3+	Heavily undulated
*G. tropicum* (Ga2)	Brown	No tearing	4+	Smooth, some undulated
*Hexagonia* sp. (He1)	Gray	Tearing on day 30, no tearing later	2+	Smooth
*Hexagonia* sp. (He2)	Brownish white	Tear easily	2+	Smooth
*Lentinus squarrosulus* (Le)	Gray	No tearing	3+	Smooth
*Microporus xanthopus* (Mi)	Gray, brown pigmentation	No tearing	3+	Smooth
*Pleurotus ostreatus* (Pl)	Brownish white	No tearing	4+	Smooth, fluffy
*Trametes polyzona* (Tr1)	White, brown pigmentation	No tearing	4+	Some undulated
*T. sanguinea* (Tr2)	Orange–red	Tear at the margin	3+	Smooth, fluffy, powdery surface

**Table 5:** Morphological features of pure mycelium mats of selected fungal strains in malt extract broth.

Fungal Strains	Colour	Rigidity	Mycelial Density	Surface Texture
*Coriolopsis brunneoleuca* (Co)	Brownish white	No tearing	3+	Smooth, fluffy
*Earliella scabrosa* (Ea1)	White with dark brown ornamentation	No tearing	4+	Undulated
*E. scabrosa* (Ea2)	White with brown ornamentation	No tearing	3+	Smooth, some undulated
*Ganoderma australe* (Ga1)	White with brownish ornamentation	No tearing	3+	Undulated
*G. tropicum* (Ga2)	White, brown upon maturity	No tearing	3+	Smooth, some undulated
*Hexagonia* sp. (He1)	Gray	No tearing	2+	Undulated
*Hexagonia* sp. (He2)	Gray, brownish upon maturity	Tearing at the margin	2+	Smooth
*Lentinus squarrosulus* (Le)	White, brownish upon maturity	No tearing	4+	Smooth, fluffy
*Microporus xanthopus* (Mi)	White	No tearing	3+	Smooth, some undulated
*Pleurotus ostreatus* (Pl)	Gray	No tearing	4+	Smooth, fluffy
*Trametes polyzona* (Tr1)	Gray with brownish ornamentation	No tearing	3+	Smooth
*T. sanguinea* (Tr2)	Light orange	Tearing at the margin	2+	Smooth, some undulated

**Table 6:** Dry density and initial moisture contents of mushroom mycelium-based biomaterial samples.

Fungal Strains	Weight Before Drying (g)	Weight After Drying (g)	Initial Moisture Content (%)	Dry Density (kg/m^3^)	Average Volumetric Shrinkage (%)
*Coriolopsis brunneoleuca* (Co)	185.74 ± 12.06 ^a^	76.49 ± 3.11 ^c^	58.76 ± 1.17 ^a^	254.97 ± 10.35 ^c^	9.52 ± 1.09% ^cd^
*Earliella scabrosa* (Ea1)	171.78 ± 6.74 ^b^	73.33 ± 0.70 ^e^	57.25 ± 1.98 ^abc^	244.44 ± 2.34 ^e^	7.13 ± 1.05% ^ef^
*E. scabrosa* (Ea2)	174.03 ± 4.08 ^b^	73.95 ± 0.35 ^de^	57.49 ± 1.04 ^abc^	246.49 ± 1.18 ^de^	8.30 ± 1.04% ^de^
*Ganoderma australe* (Ga1)	n/a *	n/a *	n/a *	n/a *	n/a *
*G. tropicum* (Ga2)	174.30 ± 4.34 ^b^	78.53 ± 2.41 ^c^	54.95 ± 0.54 ^d^	261.77 ± 8.05 ^c^	10.94 ± 1.15% ^bc^
*Hexagonia* sp. (He1)	162.86 ± 3.98 ^c^	73.26 ± 2.61 ^e^	55.01 ± 1.23 ^d^	244.21 ± 8.71 ^e^	12.73 ± 1.37% ^a^
*Hexagonia* sp. (He2)	189.75 ± 6.70 ^a^	82.16 ± 1.12 ^b^	56.65 ± 1.99 ^bcd^	273.87 ± 3.74 ^b^	7.51 ± 1.05% ^ef^
*Lentinus squarrosulus* (Le)	173.14 ± 2.25 ^b^	76.42 ± 0.88 ^c^	55.86 ± 0.24 ^cd^	254.74 ± 2.93 ^c^	11.16 ± 1.75% ^b^
*Microporus xanthopus* (Mi)	n/a *	n/a *	n/a *	n/a *	n/a *
*Pleurotus ostreatus* (Pl)	183.46 ± 4.58 ^a^	76.58 ± 0.08 ^c^	58.24 ± 1.05 ^ab^	255.27 ± 0.27 ^c^	7.60 ± 1.06% ^ef^
*Trametes polyzona* (Tr1)	169.96 ± 7.73 ^bc^	76.16 ± 1.89 ^cd^	55.09 ± 2.76 ^d^	253.87 ± 6.29 ^cd^	8.73 ± 1.64% ^de^
*T. sanguinea* (Tr2)	188.58 ± 4.40 ^a^	85.21 ± 1.69 ^a^	54.80 ± 1.07 ^d^	284.05 ± 5.62 ^a^	6.36 ± 0.00% ^f^

Values represent mean ± standard deviation. Within a column, values followed by the same superscript letter are not significantly different (*p* < 0.05) by Duncan’s multiple range test. * indicates contaminated specimens.

**Table 7:** The compressive strength, flexural strength, and impact strength of mushroom mycelium-based biomaterial samples.

Fungal Strains	Parameters					
	Compressive Strength (MPa)		Flexural Strength (MPa)		Impact Strength (kJ/m^2^)	
	95% Confidence Interval	Average	95% Confidence Interval	Average	95% Confidence Interval	Average
*Coriolopsis brunneoleuca* (Co)	0.18, 0.26	0.22 ± 0.03 ^bc^	2.16, 4.14	3.15 ± 0.80 ^a^	0.19, 0.35	0.27 ± 0.07 ^bc^
*Earliella scabrosa* (Ea1)	0.07, 0.19	0.12 ± 0.04 ^d^	1.03, 1.45	1.24 ± 0.17 ^de^	0.19, 0.41	0.30 ± 0.09 ^b^
*E. scabrosa* (Ea2)	0.10, 0.14	0.12 ± 0.02 ^d^	1.37, 1.80	1.59 ± 0.18 ^cd^	0.18, 0.34	0.26 ± 0.06 ^bc^
*Ganoderma australe* (Ga1)	n/a *	n/a *	n/a *	n/a *	n/a *	n/a *
*G. tropicum* (Ga2)	0.13, 0.27	0.20 ± 0.06 ^bc^	1.57, 2.13	1.85 ± 0.23 ^c^	0.29, 0.47	0.38 ± 0.07 ^a^
*Hexagonia* sp. (He1)	0.39, 0.48	0.44 ± 0.04 ^a^	2.52, 3.38	2.95 ± 0.34 ^a^	n/a *	n/a *
*Hexagonia* sp. (He2)	0.22, 0.27	0.25 ± 0.02 ^b^	1.27, 2.23	1.75 ± 0.38 ^c^	0.21, 0.33	0.27 ± 0.05 ^bc^
*Lentinus squarrosulus* (Le)	0.22, 0.31	0.27 ± 0.04 ^b^	2.04, 2.66	2.35 ± 0.25 ^b^	0.23, 0.30	0.26 ± 0.03 ^bc^
*Microporus xanthopus* (Mi)	n/a *	n/a *	n/a *	n/a *	n/a *	n/a *
*Pleurotus ostreatus* (Pl)	0.14, 0.20	0.17 ± 0.03 ^cd^	2.05, 2.79	2.42 ± 0.30 ^b^	0.13, 0.29	0.21 ± 0.06 ^c^
*Trametes polyzona* (Tr1)	0.24, 0.53	0.39 ± 0.12 ^a^	1.27, 2.42	1.85 ± 0.47 ^c^	0.20, 0.31	0.26 ± 0.04 ^bc^
*T. sanguinea* (Tr2)	0.17, 0.25	0.21 ± 0.03 ^bc^	0.78, 1.12	0.95 ± 0.14 ^e^	0.21, 0.24	0.23 ± 0.01 ^bc^

Values represent mean ± standard deviation. Within a column, values followed by the same superscript letter are not significantly different (*p* < 0.05) by Duncan’s multiple range test. * Specimen failed to grow.

**Table 8:** The water contact angle values of pure mycelium mat samples measured at 10 and 60 s.

	Contact Angle Value (°)			
	at 10 s		at 60 s	
Fungal Strains	95% Confidence Interval	Average	95% Confidence Interval	Average
*Coriolopsis brunneoleuca* (Co)	108.43, 132.61	120.52 ± 9.74 ^ab^	105.00, 131.12	118.06 ± 10.52 ^abc^
*Earliella scabrosa* (Ea1)	106.73, 128.93	117.83 ± 8.94 ^abc^	102.22, 123.53	112.88 ± 8.58 ^bcd^
*E. scabrosa* (Ea2)	108.11, 120.76	114.44 ± 5.10 ^bcd^	107.24, 117.38	112.31 ± 4.08 ^bcd^
*Ganoderma australe* (Ga1)	98.94, 118.46	108.7 ± 7.86 ^cd^	96.40, 114.40	105.4 ± 7.25 ^d^
*G. tropicum* (Ga2)	101.18, 114.94	108.06 ± 5.54 ^cd^	98.45, 114.27	106.36 ± 6.37 ^d^
*Hexagonia* sp. (He1)	110.14, 117.86	114 ± 3.11 ^bcd^	105.73, 115.73	110.73 ± 4.03 ^cd^
*Hexagonia* sp. (He2)	106.37, 129.82	117.94 ± 9.32 ^abc^	101.44, 123.42	112.43 ± 8.85 ^bcd^
*Lentinus squarrosulus* (Le)	112.00, 138.27	125.13 ± 10.58 ^a^	109.91, 135.86	122.88 ± 10.45 ^ab^
*Microporus xanthopus* (Mi)	114.30, 130.30	122.3 ± 6.44 ^ab^	113.45, 128.30	120.87 ± 5.98 ^abc^
*Pleurotus ostreatus* (Pl)	119.25, 131.99	125.62 ± 5.13 ^a^	116.20, 132.03	124.11 ± 6.37 ^a^
*Trametes polyzona* (Tr1)	100.93, 112.71	106.82 ± 4.74 ^d^	102.01, 107.62	104.82 ± 2.26 ^d^
*T. sanguinea* (Tr2)	116.59, 127.47	122.03 ± 4.38 ^ab^	106.98, 129.01	117.99 ± 8.87 ^abc^

Values represent mean ± standard deviation. Within a column, values with the same letter are not significantly different (*p* < 0.05) by Duncan’s multiple range test.

**Table 9:** Chemical properties (pH, organic matter content, nitrogen content and electrical conductivity) of mushroom mycelium-based biomaterials developed from selected fungal strains.

Parameters	Fungal Strains												
	Control (SD)	Co	Ea1	Ea2	Ga1	Ga2	He1	He2	Le	Mi	Pl	Tr1	Tr2
pH	7.11 ± 0.01 ^a^	4.98 ± 0.00 ^i^	5.74 ± 0.01 ^b^	5.62 ± 0.01 ^d^	n/a *	4.90 ± 0.00 ^j^	5.61 ± 0.01 ^d^	5.61 ± 0.02 ^g^	5.61 ± 0.03 ^h^	n/a *	5.61 ± 0.04 ^c^	5.61 ± 0.05 ^f^	5.61 ± 0.06 ^e^
Organic matter (%)	84.06 ± 0.25 ^a^	78.54 ± 0.91 ^cd^	76.33 ± 1.20 ^ef^	79.56 ± 0.85 ^c^	n/a *	77.79 ± 1.28 ^cde^	76.12 ± 0.63 ^ef^	81.35 ± 1.36 ^b^	76.99 ± 1.87 ^def^	n/a *	81.47 ± 0.79 ^b^	76.47 ± 1.01 ^ef^	75.06 ± 0.05 ^f^
Nitrogen (%)	0.57 ± 0.01 ^def^	0.65 ± 0.03 ^b^	0.55 ± 0.01 ^f^	0.65 ± 0.01 ^b^	n/a *	0.59 ± 0.01 ^d^	0.69 ± 0.01 ^a^	0.61 ± 0.01 ^c^	0.62 ± 0.01 ^c^	n/a *	0.56 ± 0.01 ^ef^	0.58 ± 0.00 ^de^	0.56 ± 0.01 ^ef^
Electrical conductivity (dS/m)	0.43 ± 0.00 ^j^	0.97 ± 0.01 ^d^	0.77 ± 0.00 ^h^	1.15 ± 0.01 ^c^	n/a *	1.37 ± 0.00 ^a^	0.80 ± 0.01 ^f^	1.21 ± 0.00 ^b^	0.77 ± 0.01 ^h^	n/a *	0.66 ± 0.00 ^i^	0.90 ± 0.00 ^e^	0.78 ± 0.00 ^g^

Note: Values represent mean ± standard deviation. Within a column, values with the same letter are not significantly different (*p* < 0.05) by Duncan’s multiple range test. * Specimen failed to grow.

**Table 10:** Cumulative percentage weight loss of mushroom mycelium-based biomaterials during the soil burial degradability test.

Fungal Strains	Time (Days)					
	15	30	45	60	75	90
*Coriolopsis brunneoleuca* (Co)	15.18 ± 0.44 ^bcd^	19.94 ± 0.45 ^de^	28.45 ± 2.08 ^de^	33.60 ± 2.39 ^d^	47.66 ± 7.12 ^cd^	65.17 ± 4.19 ^d^
*Earliella scabrosa* (Ea1)	14.15 ± 0.04 ^cd^	18.91 ± 0.17 ^e^	25.12 ± 2.32 ^e^	29.31 ± 1.43 ^de^	60.43 ± 4.49 ^bc^	72.87 ± 3.40 ^c^
*E. scabrosa* (Ea2)	8.78 ± 1.11 ^f^	20.13 ± 0.49 ^cde^	25.90 ± 1.47 ^e^	31.24 ± 2.86 ^de^	37.68 ± 18.94 ^de^	73.11 ± 3.30 ^c^
*Ganoderma australe* (Ga1)	n/a *	n/a *	n/a *	n/a *	n/a *	n/a *
*G. tropicum* (Ga2)	17.90 ± 0.86 ^ab^	22.44 ± 0.82 ^bcde^	24.89 ± 1.14 ^e^	26.62 ± 1.40 ^e^	29.41 ± 1.43 ^e^	32.05 ± 1.36 ^e^
*Hexagonia* sp. (He1)	15.54 ± 2.30 ^bcd^	22.47 ± 3.55 ^bcde^	30.21 ± 4.75 ^de^	41.81 ± 5.07 ^bc^	59.68 ± 5.82 ^bc^	74.63 ± 4.65 ^bc^
*Hexagonia* sp. (He2)	10.45 ± 1.53 ^ed^	23.91 ± 3.08 ^abcd^	45.17 ± 4.73 ^a^	59.08 ± 5.21 ^a^	75.16 ± 2.10 ^a^	79.70 ± 1.83 ^b^
*Lentinus squarrosulus* (Le)	12.49 ± 3.01 ^de^	27.28 ± 3.53 ^ab^	37.37 ± 3.81 ^bc^	44.57 ± 2.98 ^bc^	57.39 ± 2.94 ^bc^	73.08 ± 2.06 ^c^
*Microporus xanthopus* (Mi)	n/a *	n/a *	n/a *	n/a *	n/a *	n/a *
*Pleurotus ostreatus* (Pl)	16.90 ± 2.21 ^bc^	25.74 ± 5.43 ^ab^	47.94 ± 7.00 ^a^	56.36 ± 6.54 ^a^	69.96 ± 6.47 ^ab^	87.11 ± 2.58 ^a^
*Trametes polyzona* (Tr1)	13.49 ± 2.94 ^cde^	24.92 ± 1.16 ^abc^	34.09 ± 2.33 ^cd^	41.31 ± 3.15 ^c^	49.59 ± 2.98 ^cd^	61.36 ± 2.55 ^d^
*T. sanguinea* (Tr2)	20.65 ± 1.49 ^a^	28.45 ± 0.74 ^a^	41.35 ± 3.08 ^ab^	48.47 ± 2.84 ^b^	65.07 ± 3.22 ^ab^	72.73 ± 4.19 ^c^

Values represent mean ± standard deviation. Within a column, values followed by the same superscript letter are not significantly different (*p* < 0.05) by Duncan’s multiple range test. * Specimen failed to grow.

## Data Availability

The original contributions presented in this study are included in the article. Further inquiries can be directed to the corresponding author.

## Supplementary Materials

The following supporting information can be downloaded at: https://www.mdpi.com/article/10.3390/jof12080617/s1, Figure S1: Thermal analysis of pure mycelium mats from twelve fungal strains. (a) TGA curves; (b) DTGA curves. The different colour lines represent mycelium mats from different fungal species; Figure S2: Fourier transform infrared spectroscopy spectra of pure mycelium from selected fungal strains; Table S1: Characteristic absorption bands observed in the Fourier transform infrared spectroscopy spectra (4000–400 cm^−1^) of pure mycelium mats from selected fungal strains.
