# Supplementary material for: Characterisation of Mycelium-Based Biomaterials Derived from Diverse Mushroom Species for Sustainable Applications
Source: J Fungi (Basel). 2026 Aug 16;12(8):617. doi: 10.3390/jof12080617 (PMC13514804; doi:10.3390/jof12080617)
Supplement: Supplementary file 1 [file jof-12-00617-s001.zip › jof-4486779-supplementary.pdf]

Supplementary materials

Supplementary Figures

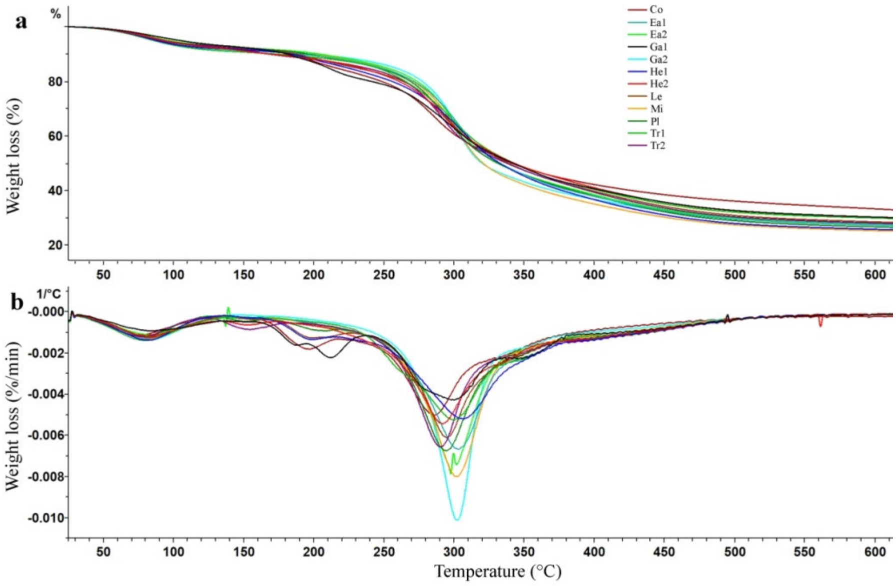

Figure S1. Thermal analysis of pure mycelium mats from twelve fungal strains. (**a**) TGA curves; (**b**) DTGA curves. The different colour lines represent mycelium mats from different fungal species.

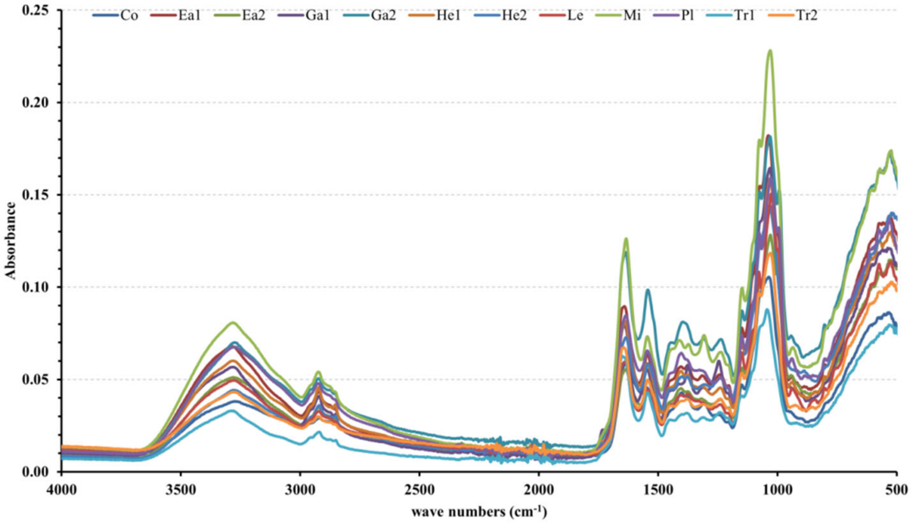

Figure S2. Fourier transform infrared spectroscopy spectra of pure mycelium from selected fungal strains.

Supplementary Table

Table S1. Characteristics absorption bands observed in the Fourier transform infrared spectroscopy spectra (4000–400cm^{-1}) of pure mycelium mats from selected fungal strains.

**Wave number (cm^{-1})**	**Band origin/Spectra**	**Wavenumbers (cm^{-1})**												**References**
		**Co**	**Ea1**	**Ea2**	**Ga1**	**Ga2**	**He1**	**He2**	**Le**	**Mi**	**Pl**	**Tr1**	**Tr2**	
3600–3000	O–H stretching hydrogen bonds	3266.54	3281.93	3281.59	3279.35	3273.32	3280.15	3280.11	3278.31	3281.55	–	3279.31	3279.88	[1,2]
3000–2840	C-H stretching in methyl and methylene groups	2920.52	2922.24	2923.03	2920.57	2924.01	2922.46, 2853.13	2921.14	2921.98	2923.89	3276.75, 2924.47	2921.52	2925.22	[3,4]
2940–2840	CH_2 asymmetric stretching	2920.52	2922.24	2923.03	2920.57	2924.01	2922.46, 2853.13	2921.14	2921.98	2923.89	2924.47	2921.52	2925.22	[5]
1658–1625	Amide I, chitin	1632.86	1640.93	1636.02	1640.75	1636.77	1638.55	1634.95	1639.35	1633.90	1635.59	1650.58	1644.51	[6]
1560–1520	C=C stretching of aromatic	1547.58	1545.29	1544.60	1546.46	1543.07	1545.66	1542.19	1544.90	1545.40	1545.09	1544.34	1543.56	[3,4]

	ring (syringyl) in lignin													
1460–1400	CH2 and CH_3 deformation in the lignin and hemicellulose, CH_2 in-plane bending vibrations in the cellulose and lignin	1402.44	–	1403.89	–	–	1404.28	1401.55	–	1412.42	1403.05	–	–	[7,8]
1390–1370	C–H symmetric deformation vibration, $R–CH_3$	–	1376.28	–	1375.37	–	–	–	1376.10	–	–	1380.44	1377.60	[9]
1375–	O-H bending	–	1315.4	–	–	–	–	–	–	–	–	–	–	[6]

1315	polysaccharide, Amide III		0											
1250–1025	C-O bond, β (1→3) glucan, cell wall polysaccharide	1149.26, 1036.37	1243.59, 1149.26, 1038.36	1238.61, 1147.98, 1074.63, 1029.34	1245.96, 1149.67, 1031.25	1238.33, 1148.21, 1074.96, 1030.61	1239.51, 1149.66, 1076.23, 1030.47	1239.45, 1032.44	1238.92, 1149.44, 1077.42, 1025.98	1236.04, 1148.12, 1075.71, 1030.30	1236.70, 1149.24, 1076.79, 1027.71	1243.23, 1043.14	1239.05, 1149.47, 1076.66, 1029.59	[6]
1162–1125	C-O-C vibration in cellulose and hemicellulose	1149.26	1149.26	1147.98	1149.67	1148.21	1149.66	–	1149.44	1148.12	1149.24	–	1149.47	[1,3,4]
1060–1015	C-O valence vibration from C3-O3H	1036.37	1038.36	1029.34	1031.25	1030.61	1030.47	1032.44	1025.98	1030.30	1027.71	1043.14	1029.59	[4]
1047–1004	C-O stretching in	1036.37	1038.36	1029.34	1031.25	1030.61	1030.47	1032.44	1025.98	1030.30	1027.71	1043.14	1029.59	[1,3,4]

	cellulose													
1185–900	Polysaccharides	1149.26, 1036.37	1149.26, 1038.36	1147.98, 1074.63, 1029.34, 992.85	1149.67, 1031.25	1148.21, 1074.96, 1030.61, 992.62	1149.66, 1076.23, 1030.47, 939.08	1032.44	1149.44, 1077.42, 1025.98, 938.13	1148.12, 1075.71, 1030.30, 935.52	1149.24, 1076.79, 1027.71, 994.77,	1043.14	1149.47, 1076.66, 925.47	[10]
950–700	glycosidic bond β-(1 → 4) arabinogalactans, galactomannans, and cellulose	–	–	803.00	–	–	939.08	–	938.13	935.52	939.66	–	925.47	[11]

References:

1. Mohebby, B. Attenuated total reflection infrared spectroscopy of white-rot decayed beech wood. *Int. Biodeterior. Biodegrad.* **2005**, *55*, 247–251.
2. Haneef, M.; Ceseracciu, L.; Canale, C.; Bayer, I.S.; Heredia-Guerrero, J.A.; Athanassiou, A. Advanced materials from fungal mycelium: fabrication and tuning of physical properties. *Sci. Rep.* **2017**, *7*, 41292.
3. Pandey, K.K.; Pitman, A.J. FTIR studies of the changes in wood chemistry following decay by brown-rot and white-rot fungi. *Int. Biodeterior. Biodegrad.* **2003**, *52*, 151–160.
4. Schwanninger, M.; Rodrigues, J.C.; Pereira, H.; Hinterstoisser, B. Effects of short-time vibratory ball milling on the shape of FT-IR spectra of wood and cellulose. *Vib. Spectrosc.* **2004**, *36*, 23–40.
5. Elsacker, E.; Vandelook, S.; Brancart, J.; Peeters, E.; De Laet, L. Mechanical, physical and chemical characterisation of mycelium-based composites with different types of lignocellulosic substrates. *PLoS ONE* **2019**, *14*, e0213954.
6. D'Souza, R.A.; Kamat, N.M. Potential of FTIR spectroscopy in chemical characterization of *Termitomyces* pellets. *J. App. Biol. Biotech.* **2017**, *5*, 80–84.
7. Raspolli Galletti, A.M.; D'Alessio, A.; Licursi, D.; Antonetti, C.; Valentini, G.; Galia, A.; Nassi O Di Nasso, N. Midinfrared FT-IR as a tool for monitoring herbaceous biomass composition and its conversion to furfural. *J. Spectrosc.* **2015**, 1–12.
8. Akcay, C.; Ceylan, F.; Arslan, R. Production of oyster mushroom (*Pleurotus ostreatus*) from some waste lignocellulosic materials and FTIR characterization of structural changes. *Sci. Rep.* **2023**, *13*, 12897.
9. Baudot, C.; Tan, C.M.; Kong, J.C. FTIR spectroscopy as a tool for nano-material characterization. *Infrared Phys. Technol.* **2010**, *53*, 434–438.
10. Naumann, A. A novel procedure for strain classification of fungal mycelium by cluster and artificial neural network analysis of Fourier transform infrared (FTIR) spectra. *Analyst* **2009**, *134*, 1215.
11. Reis, N.; Botelho, B.G.; Franca, A.S.; Oliveira, L.S. Simultaneous detection of multiple adulterants in ground roasted coffee by ATR-FTIR spectroscopy and data fusion. *Food Anal. Methods* **2017**, *10*, 2700–2709.
